# Overexpression of differentially expressed *AhCytb6* gene during plant-microbe interaction improves tolerance to N_2_ deficit and salt stress in transgenic tobacco

**DOI:** 10.1038/s41598-021-92424-4

**Published:** 2021-06-28

**Authors:** Ankita Alexander, Vijay K. Singh, Avinash Mishra

**Affiliations:** 1 Division of Applied Phycology and Biotechnology, CSIR-Central Salt and Marine Chemicals Research Institute, G. B. Marg, Bhavnagar, Gujarat 364002 India; 2 Academy of Scientific and Innovative Research (AcSIR), CSIR, Ghaziabad, India; 3 Present Address: Department of Microbiology, Harvard Medical School, Boston, MA USA; 4 Present Address: Department of Surgery, Massachusetts General Hospital, Boston, MA USA

**Keywords:** Molecular engineering in plants, Abiotic

## Abstract

*Stenotrophomonas maltophilia* has plant growth-promoting potential, and interaction with *Arachis hypogaea* changes host-plant physiology, biochemistry, and metabolomics, which provides tolerance under the N_2_ starvation conditions. About 226 suppression subtractive hybridization clones were obtained from plant-microbe interaction, of which, about 62% of gene sequences were uncharacterized, whereas 23% of sequences were involved in photosynthesis. An uncharacterized SSH clone, SM409 (full-length sequence showed resemblance with *Cytb6*), showed about 4-fold upregulation during the interaction was transformed to tobacco for functional validation. Overexpression of the *AhCytb6* gene enhanced the seed germination efficiency and plant growth under N_2_ deficit and salt stress conditions compared to wild-type and vector control plants. Results confirmed that transgenic lines maintained high photosynthesis and protected plants from reactive oxygen species buildup during stress conditions. Microarray-based whole-transcript expression of host plants showed that out of 272,410 genes, 8704 and 24,409 genes were significantly (*p* < 0.05) differentially expressed (> 2 up or down-regulated) under N_2_ starvation and salt stress conditions, respectively. The differentially expressed genes belonged to different regulatory pathways. Overall, results suggested that overexpression of *AhCytb6* regulates the expression of various genes to enhance plant growth under N_2_ deficit and abiotic stress conditions by modulating plant physiology.

## Introduction

Plant growth-promoting rhizobacteria (PGPR) improve plant growth and development directly and/or indirectly: directly by nitrogen fixation, phosphate solubilization, siderophore, and phytohormone production and indirectly by acting as a biocontrol agent or by activating induced systemic resistance (ISR) in the host plant^1,2^. Interaction of PGPR or pathogenic bacteria with the host plants causes various signalings, which leads to the activation of the host immune system. However, plants differentiate between PGPR and pathogenic bacteria based on response times, activation of genes, and their expression levels^3,4^. Utilization of the potential of the PGPR and their effect on the host transcriptional machinery is a great avenue for the development of sustainable agriculture for biotic and abiotic stress-affected crop plants.

According to the Intergovernmental Panel on Climate Change^5^, changes in climatic conditions and agricultural habits, and increased use of chemical fertilizers cause various abiotic stresses in legumes, which affect their growth and productivity. Abiotic stresses in soil (salt, cold, drought, waterlogging, metal toxicity, pH, and low availability of nutrients, among others) cause an alteration in the microbial flora of soil, which affects the symbiotic relationship between legumes and rhizobia^6,7^. Nitrogen is a major nutrient element for plant growth and development due to its central role and presence in many biomolecules like protein, chlorophyll, and nucleic acid^8^. Nitrogen also acts as a regulator for the carbon cycle, which directly affects the photosynthetic machinery of plants^9^. It is well established that nutrient homeostasis plays a key role in plant growth and development. Nutrient deficiency, including the N_2_ starvation condition, leads to stress conditions and activates the nutrient-deprivation signal transduction. In nitrogen starvation conditions, plants use their stored nitrogen, and more than half of the leaf nitrogen is used in photosynthetic machinery, thus plants have to compromise with growth (less nitrogen for structural proteins) and yield (early senescence)^10^. Differential expression of key genes coordinates with plant physiology to manage the demand for nutrients. The low availability of nitrogen in the soil decreases the yield of the crop, which could be compensated for by the application of N_2_ fixing bacteria^11^. In this scenario, we need diazotrophic bacteria that are tolerant to abiotic stress and act as PGPR to balance the nutrient cycle between the plant-microbe-soil dynamic in stress conditions. The use of PGPR for the enhancement of crop productivity under various biotic and abiotic stresses is better for sustainable and environmentally friendly agriculture^12^.

There are a plethora of studies that show the improvement in yield and health of plants after application of PGPR^13–16^, and some studies showed changes at the molecular level (transcript expression) in the host plant after interaction with PGPR^17^. There is a need to understand the changes and events taking place at the molecular level in the host plant after interaction with PGPR under abiotic stress, and utilizing the differentially expressed gene for the potential candidate for the bioengineering of the host genome could be a highly translational strategy.

In nature, the peanut plant is associated with various nitrogen-fixing, nodulating rhizobacteria, which help in nitrogen fixation. However, the lack of specificity of this interaction make it difficult to understand the specific changes that occur at the molecular level during the interaction. To understand the effect of a specific single PGPR on plant growth promotion and molecular changes in *A. hypogaea*, we used the strain *Stenotrophomonas maltophilia* BJ01, isolated from non-crop and non-leguminous plants from the coastal saline area^18^. We reported the effect of single PGPR *S. maltophilia* BJ01 on the physio-biochemical and metabolic changes on the host plant under nitrogen starvation and salt stress conditions^19,20^. Differential expression of genes due to plant-PGPR interaction will provide the molecular mechanism of PGPR-action as well as useful insight about the potential gene candidates to be explored for sustainable agriculture under stress conditions.

In this study, we found that the *AhCytb6* gene is differentially expressed in peanut under nitrogen-starved conditions after interaction with *S. maltophilia* BJ01. To understand the role of this gene in the host plant, we engineered the genome of the model plant (tobacco) and inserted *AhCytb6* along with the expression cassette in the genome. Ectopic overexpression of the *AhCytb6* gene in transgenic tobacco enhances plant performances under nitrogen starvation and salt stress. The role of *AhCytb6* was also explored for the growth and development of plants and their stress responses. *Cytb6* is a key regulatory unit of the electron transport chain in plants and affects the photosynthetic efficiency and yield of plants^21,22^. Recently, Lande et al. reported that abiotic stress drastically decreases the proteins related to *Cytb6* in chickpea^23^. Overexpression of this gene increases the photosynthetic ability, biomass and yield of the plants under various abiotic stresses^24–26^. Our results showed that the photosynthesis gene *AhCytb6* is differentially expressed in host plants due to interaction with PGPR, and overexpression of this gene provides tolerance to the model plants under N_2_ starvation and abiotic stress conditions.

## Results

### Differential expression of genes in the response of ***S***. ***maltophilia*** BJ01 under N_2_ starvation condition

**Figure 1:**
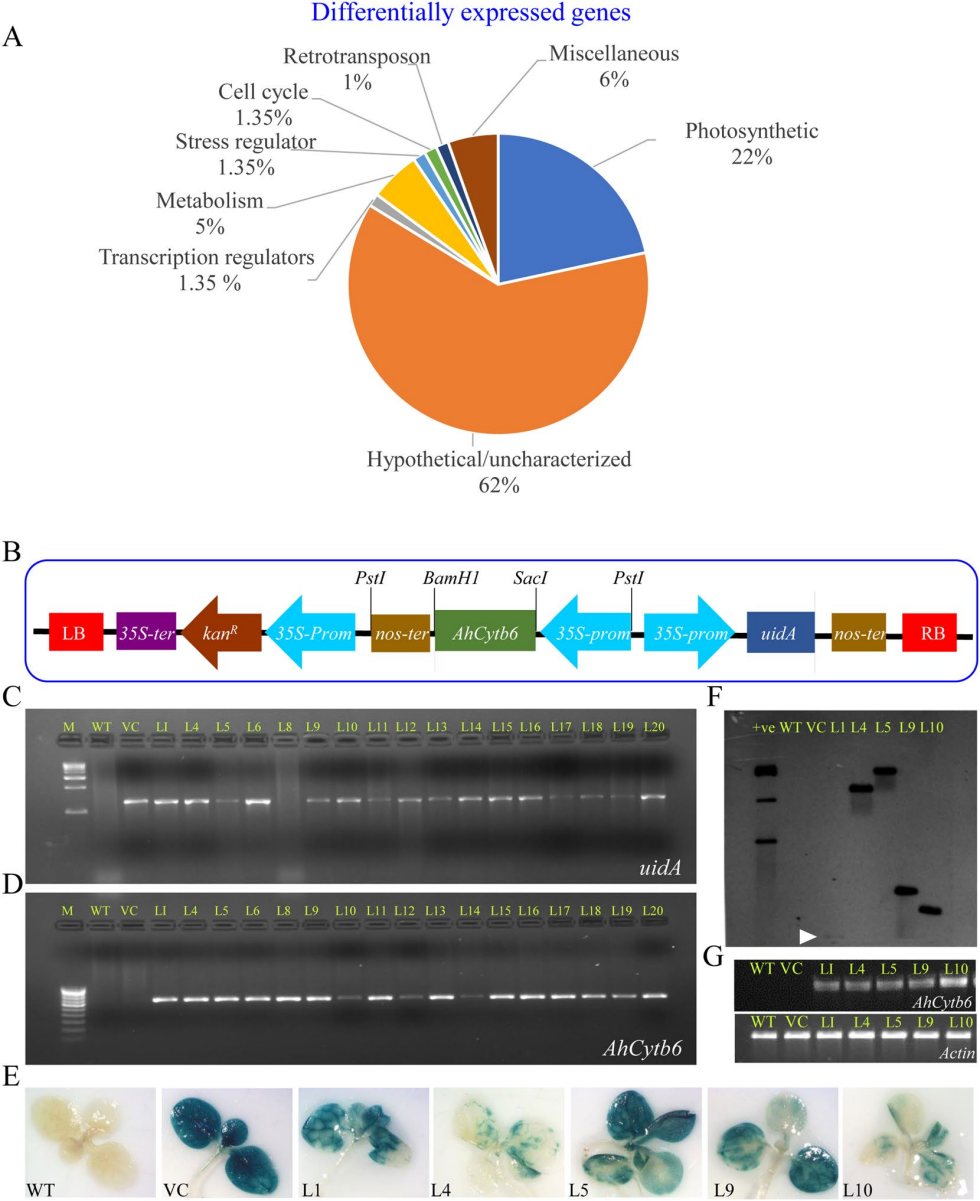
Differential expression of genes analyzed by suppression subtractive hybridization and genetic transformation and molecular confirmation of transgenic lines. (**A**) Genes were differentially expressed in *Arachis hypogaea* treated with plant growth promoting *S. maltophilia* BJ01 plant under N_2_ starvation condition (**B**) Schematic representation of *AhCytb6* gene-pCAMBIA2301 plant transformation vector construct, PCR amplification of (**C**) *uidA* and (**D**) *AhCytb6* genes, (**E**) Histochemical GUS assay of selected transgenic lines, (**F**) Southern blot and (**G**) Reverse transcriptase PCR analysis.

There were 400 SSH (suppression subtractive hybridization) clones sequenced and subjected to chimera analysis, and 226 resultant clone sequences were obtained, which were subjected to BLAST and categorized into eight-groups (Fig. 1A). Interestingly, about 62% of differentially expressed gene sequences did not show significant similarity with known genes and were categorized as unknown/uncharacterized/hypothetical. Similarly, 23% of EST sequences were involved in photosynthesis. About 3% of sequences belonged to apoptosis, while 1% EST were signaling molecules, transcription factors, stress regulators, and metabolism. About 3% of sequences did not show any resemblance and fell under the miscellaneous category. Transcript profiling of representative genes from selected categories showed differential up-regulation in PGPR-treated peanut plants (Fig. S1). Based on transcript expression profiling, clone SM409 showed 4.1-fold upregulation and resemblance with uncharacterized/ hypothetical protein and was selected for further study. The full-length SM409 clone (ORF) sequence showed resemblance (99.69% sequence similarity with 100% query coverage) to the chloroplast genome (CDS: cytochrome b6) of *Arachis spp.*, especially different cultivars of *Arachis hypogaea* (accession no. *CP030984*; *MG814006–9*; *NC_037358*; *KX257487*; *KJ468094*); therefore, the cloned gene was named *AhCytb6*. Moreover, the deduced protein (amino acid) sequence showed 93.72% similarity (with 97% query coverage) with the cytochrome b6 protein of *Arachis hypogaea* (accession no. *YP_009472186*) in the homology search*.*

### Cloning and *in silico* analysis of the *AhCytb6* gene

The *AhCytb6* gene was 1287 base pair long (accession no. *MT395343*) and was comprised of 34 bp 5′-untranslated leader sequences (5′-UTR), 636 bp of an open reading frame (ORF) and 617 bp of a 3′-UTR (5′-UTR: 1–34 bp, ORF: 35–670 bp and 3′-UTR: 671–1287 bp) (Fig. S2). In genome organization study, the amplification of 636 bp *AhCytb6* gene ORF was obtained from both genomic and cDNA, which revealed that the gene is intronless (Fig. S3). The ORF encodes for 211 amino acids having a molecular mass of 23.59 kD. *In silico* analysis revealed that the *PI* of the deduced protein was 10.6 and the instability index was 32.98; the protein half-life was predicted 30 h in mammalian reticulocytes (*in vitro*), more than 20 h in yeast (*in vivo*), and more than 10 h in *Escherichia coli* (*in vivo*), which showed that the protein was stable in nature. The *in silico* analysis predicted that the *Ah*Cytb6 peptide contained four transmembrane domains and was in the plasma membrane (Fig. S4).

### Genetic transformation and molecular confirmation of transgenic lines

About 25 putative transgenic lines (T0) were obtained after tissue culture, out of which 17 lines showed seed germination on kanamycin, which carried forward further for the generation of T1 transgenic lines. Integration transgenes were confirmed in all 17-transgenic lines by amplification of 1.2 kb of the *uidA* gene and 636 bp of the *AhCytb6* gene (Fig. 1B–D and Fig. S5). All plants were found positive, and based on histochemical *gus* expression, five lines (L1, L4, L5, L9, and L10) were selected (Fig. 1E). Selected transgenic lines showed single gene integration and high expression of the *AhCytb6* gene analyzed by southern blot and semi-quantitative RT-PCR analysis, respectively, in all selected lines (Fig. 1F, G and Fig. S5).

### Overexpression of *AhCytb6* gene enhances the growth of transgenic under N_2_ starvation and salt stress

**Figure 2:**
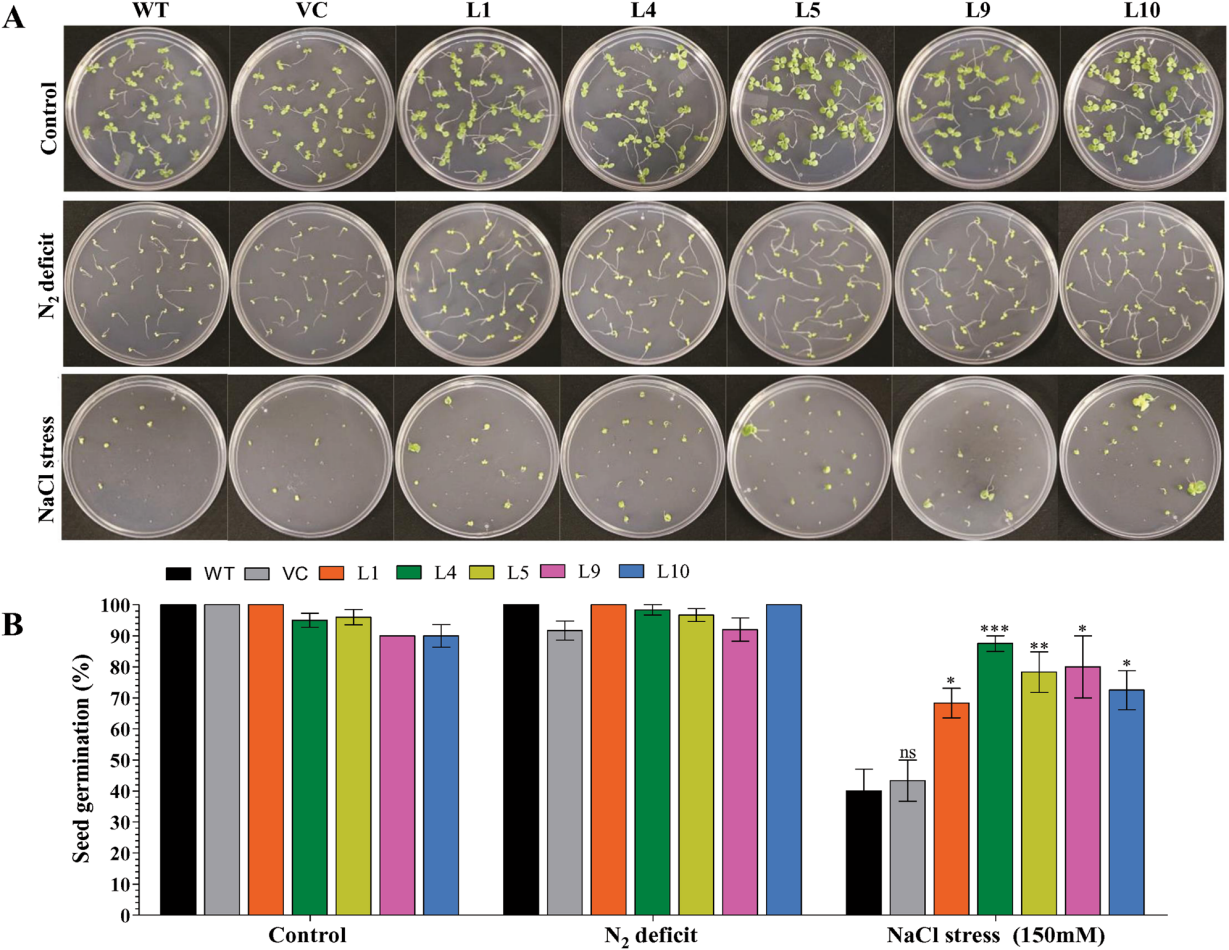
Analysis of seed germination of transgenic plants. Comparative analysis of (**A**) seed germination efficiency and (**B**) seed germination percentage of selected transgenic lines, WT and VC plants under control, N_2_ deficit and salt stress condition. Bars represent means ± standard error, and ‘*’, ‘**’ and ‘***’ designates for significant differences at P < 0.05, P < 0.01 and P < 0.001, respectively and ‘ns’ represents no significant difference.

About 100% seed germination was observed under control (unstressed) conditions, and similar results were also found for the N_2_ starvation condition. However, the percent of seed germination decreased under salt stress. About 40–42% of WT and VC seeds germinated, whereas 65–80% seed germination was estimated for transgenic (L1, L4, L5, L9, and L10) lines (Fig. 2A, B). Results suggested that the N_2_ starvation condition did not affect germination, while salt stress severely affects seed germination. Further, the overexpression of the *AhCytb6* gene enhanced the seed germination efficiency of transgenic plants under salt stress conditions compared to WT and VC plants.

**Figure 3:**
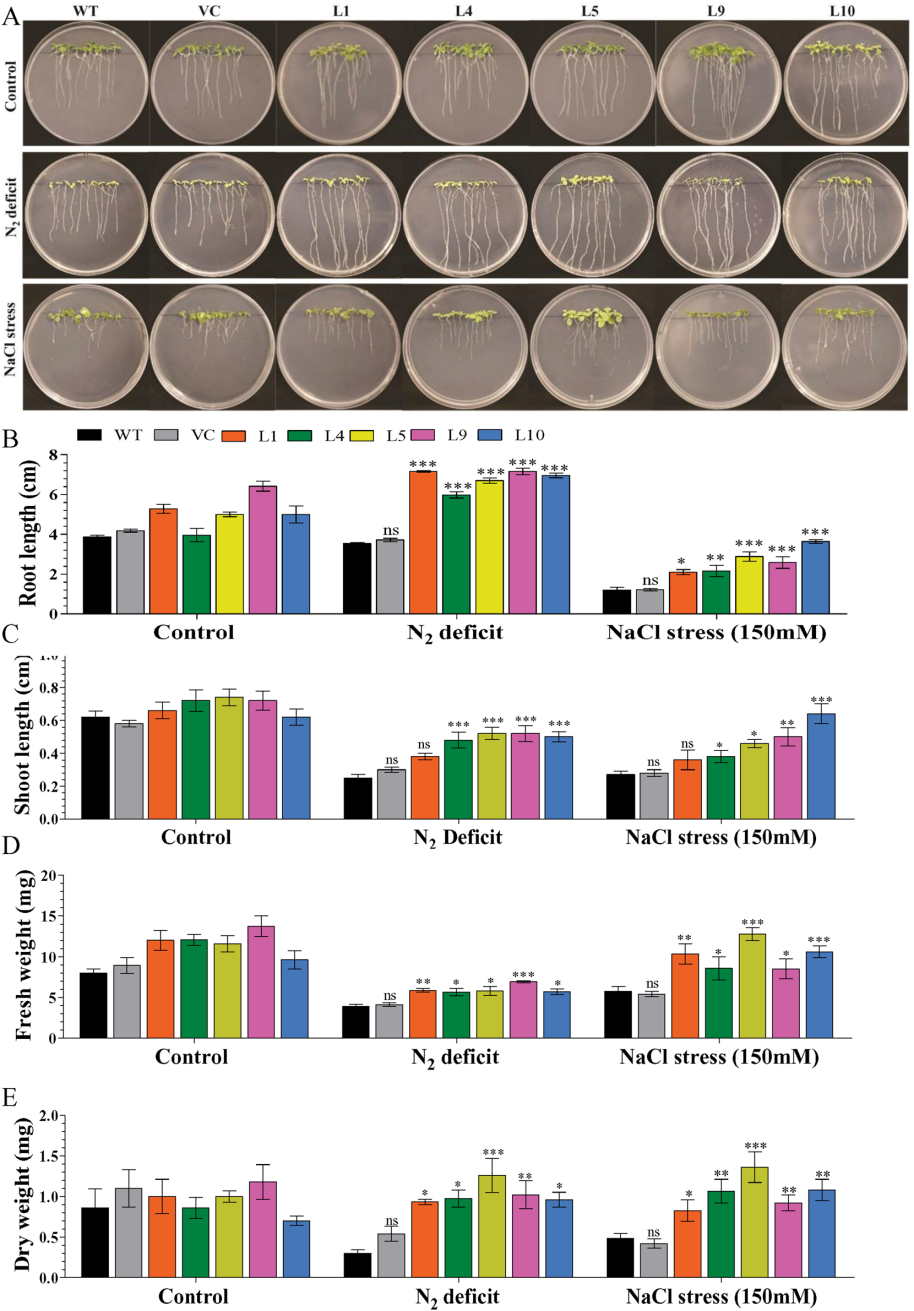
Analysis of plant growth of transgenic plants. (**A**) Comparative analysis of seedling growth of selected transgenic lines and control plants under N_2_ deficit and salt stress condition. (**B**) Root length, (**C**) shoot length, (**D**) fresh weight and (**E**) dry weight of selected transgenic lines, WT and VC plants under control, N_2_ deficit and salt stress condition. Bars represent means ± standard error, and ‘*’, ‘**’ and ‘***’ designates for significant differences at P < 0.05, P < 0.01 and P < 0.001, respectively and ‘ns’ represents no significant difference.

Enhanced plant growth of transgenic plants (L1, L4, L5, L9, and L10) was observed under stress conditions compared to WT and VC plants (Fig. 3). About 6–7 cm root length (RL), 0.4–0.5 cm shoot length (SL), 6–7 mg fresh weight (FW), and 0.9–1.3 mg dry weight (DW) were estimated in transgenic lines compared to WT and VC plants (RL: 3–4 cm, SL: 0.2–0.3 cm, FW: 3.9–4.1 mg, and DW: 0.3–0.5 mg) under N_2_ deficit stress conditions. Similarly, higher growth parameters (RL: 2–4 cm, SL: 0.4–0.6 cm, FW: 9–12 mg, and DW: 0.7–1.4 mg) were measured in transgenic plants compared to WT and VC plants (RL: 1–1.2 cm, SL: 0.27–0.28 cm, FW: 5–7 mg, and DW: 0.4–0.5 mg) under salt stress conditions (Fig. 3A–E).

### The physiological status of the transgenic plant is modulated by the ectopic expression of the *AhCytb6* gene

**Figure 4:**
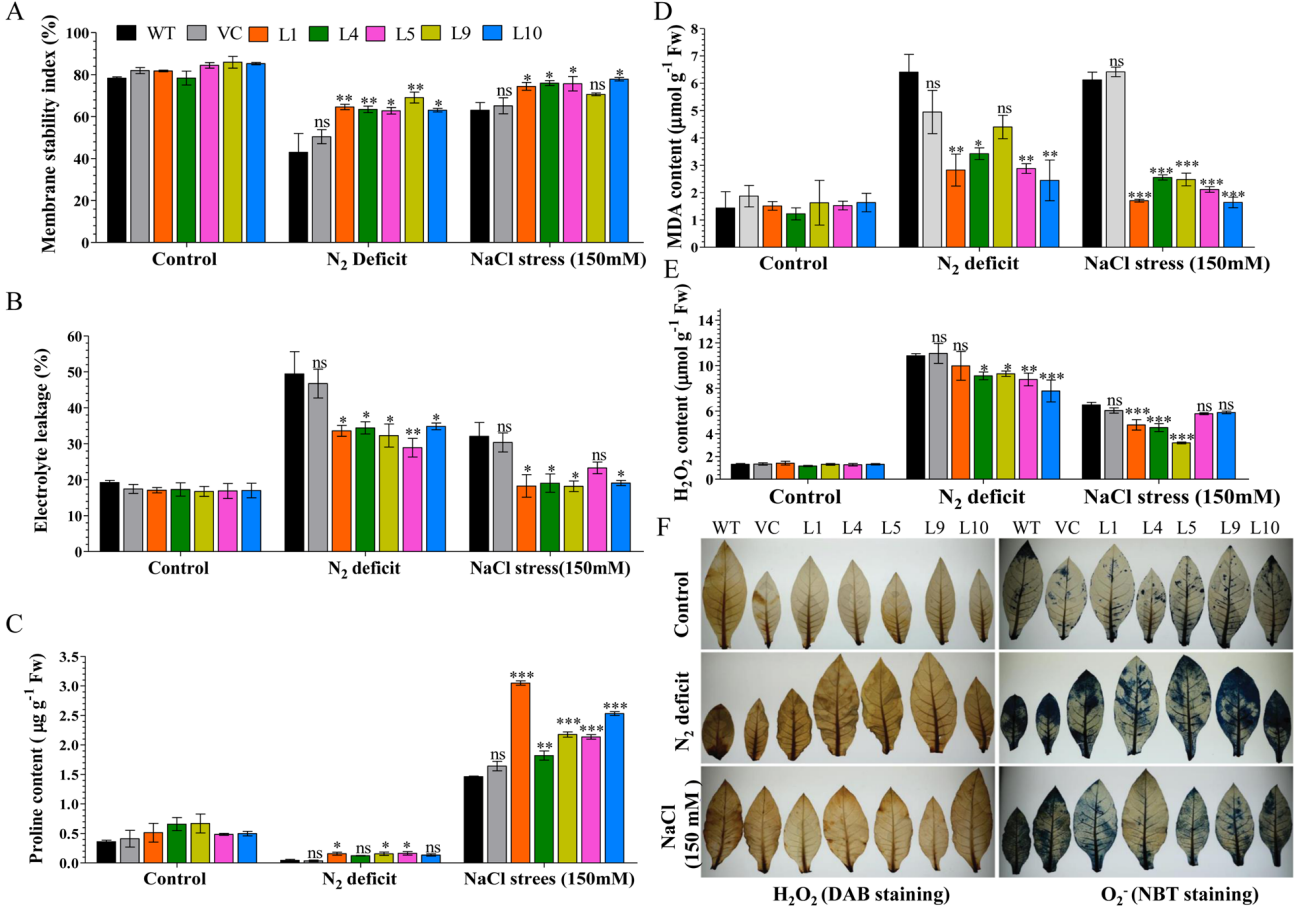
Physiological status and ROS analysis of transgenic plants. Comparative analysis of (**A**) membrane stability, (**B**) electrolyte leakage and (**C**) proline accumulation (**D**) lipid peroxidation (MDA content), (**E**) H_2_O_2_ content and (**F**) *in vivo* localization of ROS in transgenic lines, WT and VC plants under control, nitrogen deficit and salt stress condition. Bars represent means ± standard error, and ‘*’, ‘**’ and ‘***’ designates for significant differences at P < 0.05, P < 0.01 and P < 0.001, respectively and ‘ns’ represents no significant difference.

Improved membrane stability and low electrolyte leakage were found in transgenic lines (L1, L4, L5, L9, and L10) compared to WT and VC plants under stress conditions (Fig. 4A, B). About 29–35% electrolyte leakage was found in transgenic lines, which were considerably lower than WT and VC plants (47–49%) under N_2_ deficit conditions. Similarly, lower electrolyte leakage, about 18–23%, was observed in transgenic lines compared to WT and VC plants (30–32%) under salt stress conditions. High membrane stabilities, about 63–69%, and 71–78%, were estimated for transgenic lines under N_2_ deficit and salt stress conditions, respectively, compared to WT and VC plants (43–50% and 63–65%, respectively). Accumulation of proline, a common physiological response indicator, and a key player in plant abiotic stress tolerance was observed in transgenic plants in N_2_ deficit and salt stress conditions compared to WT and VC plants (Fig. 4C). Under control conditions, a similar level of proline contents was observed in transgenic lines as well as WT and VC plants. Under N_2_ deficit conditions, proline contents were about 0.12–0.16 µg g^−1^ Fw in transgenic plants and about 0.035–0.045 µg g^−1^ Fw in WT and VC. Under salt stress conditions, about 1.8–3.0 µg g^−1^ Fw of proline contents were detected in transgenic lines and about 1.4–1.6 µg g^−1^ Fw in WT and VC plants.

### The *AhCytb6* gene protects the plant from ROS buildup during stress conditions

Under control conditions, lipid peroxidation and H_2_O_2_ contents were similar in control and transgenic plants. Under N_2_ deficit condition, transgenic lines (L1, L4, L5, L9, and L10) showed significantly lower production of MDA (2.5–4.5 µmol g^−1^ Fw) and H_2_O_2_ (8–10 µmol g^−1^ Fw) in comparison to WT and VC (5–7 µmol g^−1^ Fw MDA and 11 µmol g^−1^ Fw H_2_O_2_) plants. Similarly, transgenic lines showed significantly lower accumulation of MDA (1.5–2.5 µmol g^−1^ Fw) and H_2_O_2_ (5–7 µmol g^−1^ Fw) under salt stress condition compared to WT and VC (6–6.5 µmol g^−1^ Fw MDA and 7 µmol g^−1^ Fw H_2_O_2_) plants (Fig. 4D, E). The above results were further supported by lower *in vivo* localization of H_2_O_2_ and O_2_^-^ in transgenic leaves compared to their WT and VC counterparts under stress conditions (Fig. 4F).

### Ectopic expression of the *AhCytb6* gene increases the photosynthesis efficiency under stress conditions

**Figure 5:**
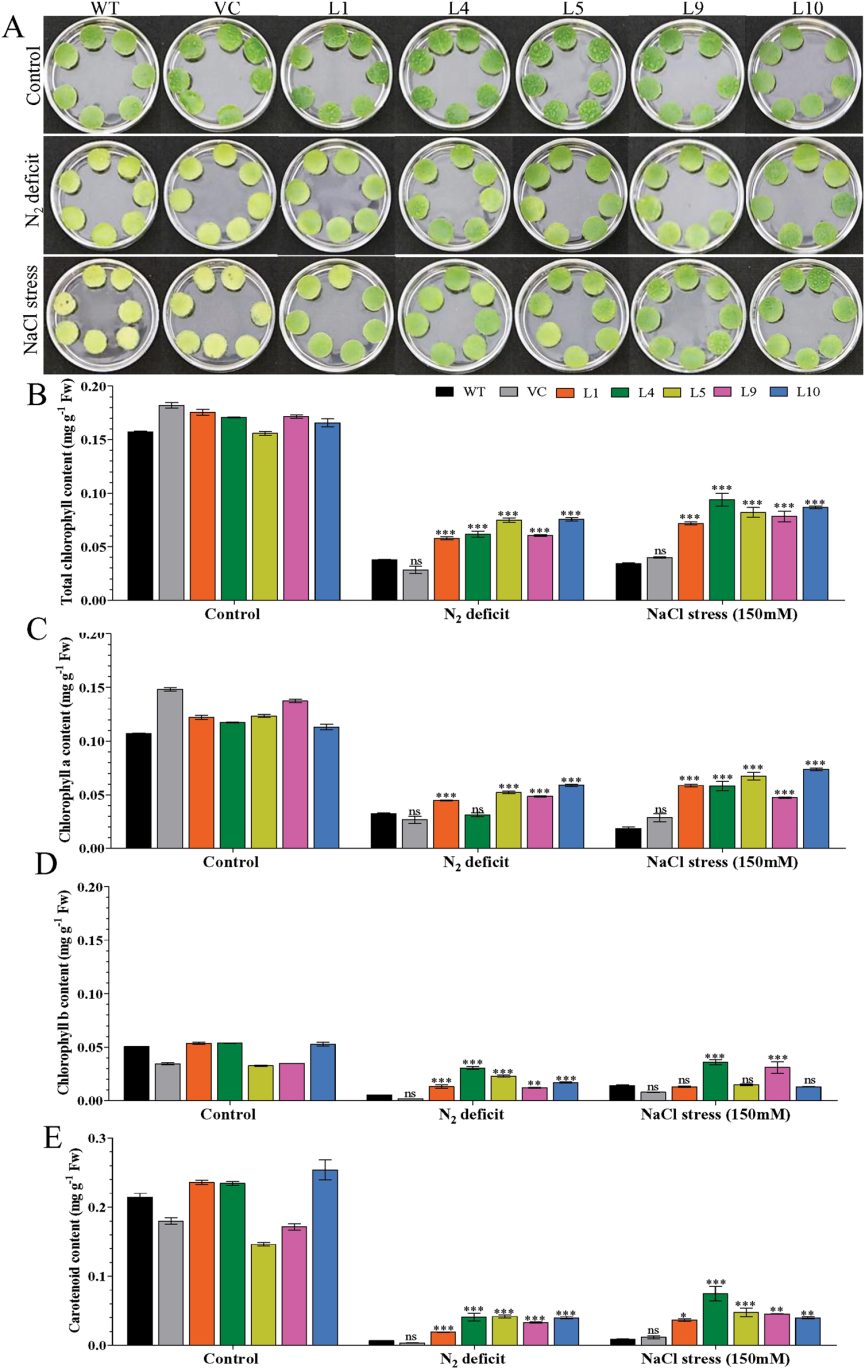
Photosynthesis efficiency analysis of transgenic plants. Comparative analysis of (**A**) leaf senescence and photosynthetic pigments, (**B**) total chlorophyll, (**C**) chlorophyll a, (**D**) chlorophyll b, (**E**) carotenoid in transgenic lines, WT and VC plants under control, nitrogen deficit and salt stress condition. Bars represent means ± standard error, and ‘*’, ‘**’ and ‘***’ designates for significant differences at P < 0.05, P < 0.01 and P < 0.001, respectively.

**Figure 6:**
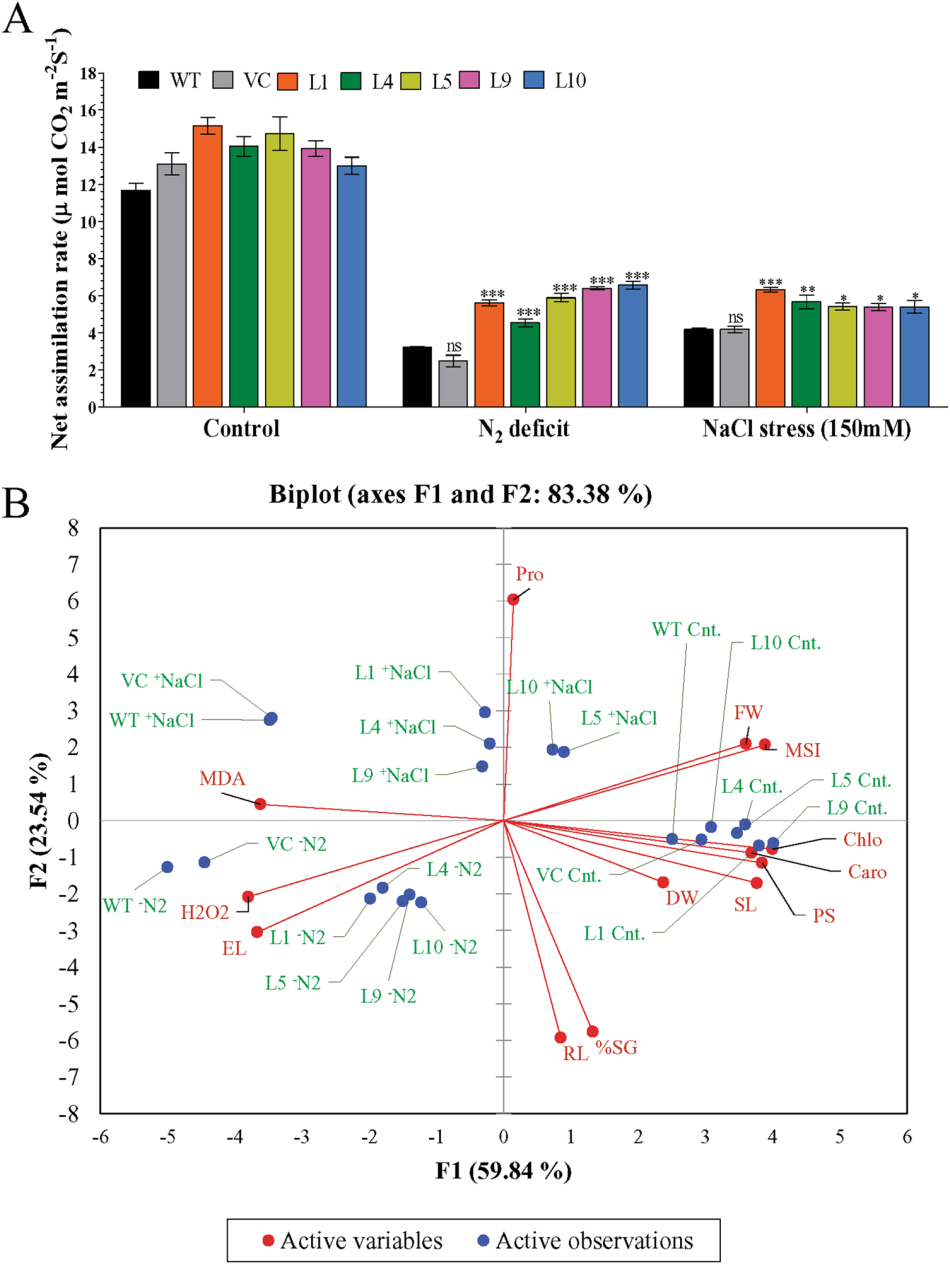
Photosynthesis efficacy and the principal component analysis of transgenic plants. Comparative analysis of (**A**) net assimilation in transgenic lines and WT and VC plants under control, nitrogen deficit and salt stress condition, (**B**) Bi-plot based principal component analysis with first two principal components showing the differential response of transgenic lines and WT plants under un-stress and stress conditions. Bars represent means ± standard error, and ‘*’, ‘**’ and ‘***’ designates for significant differences at P < 0.05, P < 0.01 and P < 0.001, respectively.

The leaf senescence assay showed a major loss of photosynthetic pigments (bleaching of leaf discs) in WT and VC plants under stress conditions compared to transgenic (L1, L4, L5, L9, and L10) lines (Fig. 5A). Higher contents of total chlorophyll (0.05–0.07 mg g^−1^ Fw), chlorophyll a (0.03–0.05 mg g^−1^ Fw), chlorophyll b (0.01–0.03 mg g^−1^ Fw), and carotenoids (0.01–0.04 mg g^−1^ Fw) were estimated in transgenic lines under N_2_ deficit conditions compared to WT and VC (total chl: 0.02–0.03; chl a: 0.02–0.03; chl b: 0.001–0.005 and carotenoids: 0.003–0.006 mg g^−1^ Fw) plants. Similarly, higher contents of photosynthesis pigments (total chl: 0.07–0.09; chl a: 0.05–0.07; chl b: 0.01–0.04 and carotenoids: 0.03–0.07 mg g^−1^ Fw) were found in transgenic lines under salt stress conditions compared to WT and VC (total chl: 0.03–0.04; chl a: 0.01–0.03; chl b: 0.008–0.01 and carotenoids: 0.009–0.01 mg g^−1^ Fw) plants (Fig. 5B–E). The net photosynthesis of transgenic plants was higher under the stress (5–7 µmol CO_2_ m^−2^ s^−1^) environment compared to WT and VC (2–4 µmol CO_2_ m^−2^ s^−1^) plants (Fig. 6A). Similarly, stomatal conductance (0.02–0.05 mol H_2_O m^−2^ s^−1^) and transpiration rate (1–1.5 mmol H_2_O m^−2^ s^−1^) were also higher in transgenic compared to WT and VC plants under stress conditions, but it was not significant (Fig. S6). Results confirmed that transgenic plants (L1, L4, L5, L9, and L10) maintained high photosynthesis under stress conditions compared to WT and VC plants.

### Multivariate analysis of morphological, biochemical, and physiological responses of plants

Principal component analysis (PCA) was performed to distinguish the different responses of transgenic and control plants under normal and stress conditions (Fig. 6B). A bi-plot inferred from the PCA separated plant responses in the first two-component with overall 83.38% variability (PC1: 59.84% and PC2: 23.54%). All plants (transgenic lines and control) showed comparable morphological, biochemical, and physiological responses in the unstressed conditions, as transgenic and WT plants clustered together (cnt) in the bi-plot analysis. Transgenic lines exhibited a differential response to the varying stresses. Among different stress conditions, plants responded towards EL and H_2_O_2_ accumulation under N_2_ deficit conditions compared to salinity stress. Similarly, plants responded further for lipid peroxidation, analyzed by MDA quantification, under salt stress compared to N_2_ deficit condition. Transgenic lines L5 and L10 were inclined towards proline accumulation under salt stress compared to other lines.

### Transcriptional regulation of transgenic tobacco by the *AhCytb6* gene under stress condition

**Figure 7:**
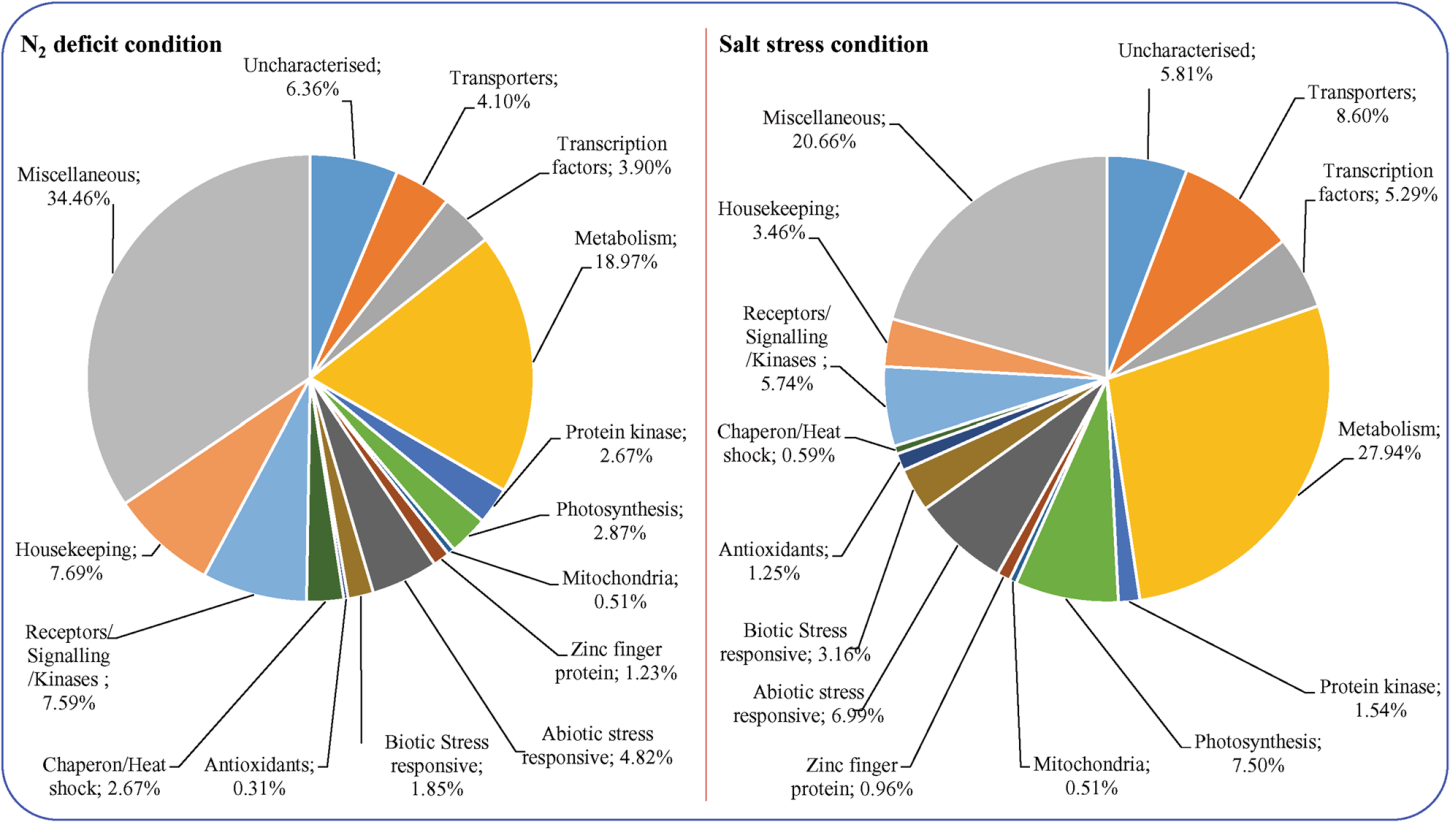
Microarray-based functional classification of host stress responsive genes. Functional classification of differentially expressed genes of *AhCytb6* overexpressing transgenic tobacco plant under abiotic stress conditions. Genes differentially expressed in the *AhCytb6* plant under stress conditions were normalized with the transcript of WT plants treated with the same stress.

The effect of the overexpression of the *AhCytb6* gene on the whole-transcript expression of the host plant was studied under stress conditions (N_2_ deficit and salt stress) using microarray (ArrayExpress ID *E-MTAB-9307*). The differential expression profiling of 272,410 gene-probes was done, and hierarchical cluster analysis, as well as scatter plots, were analyzed (Fig. S7). The analysis showed out of 272,410 genes, 8,704 and 24,409 genes were significantly (*p* < 0.05) differentially expressed (> 2 up or down-regulated) under N_2_ starvation and salt stress conditions, respectively. However, at a 4-fold change level (> 4 up or down-regulated), a total of 975 genes were differentially expressed, with 611 genes up-regulated and 364 genes down-regulated in the treated transgenic plant compared to WT under N_2_ deficit conditions. Similarly, 1360 genes were differentially expressed in the treated transgenic plant compared to WT under salt stress conditions at a 10-fold change level, out of which 1115 genes were up-regulated while 245 genes were down-regulated (Fig. 7 and Fig. S8). Some of the important differentially expressed genes are listed and grouped into different categories based on their biological activity in Table 1.

**Table 1:** Selected transcripts that differentially expressed (up- or down-regulated) in *AhCytb6* overexpressing transgenic tobacco plant compared with the wild type under nitrogen deficit or salt stress conditions.

**S. no.**	**Transcript probe ID**	**Gene name**	**Gene accession**	**Fold change (log2)**
**Transcripts significantly differentially expressed under nitrogen deficit stress condition**				
**Photosynthesis**				
1.	NtPMIa1g85328e1_st	RuBisCO large subunit-binding protein	FH655192	4.36
2.	NtPMIa1g189087e1_st	Rubredoxin RubB	FH744456	3.88
3.	NtPMIa1g145438e1_st	Rubisco accumulation factor 2	FH522410	3.72
4.	NtPMIa1g46069e1_st	Cytochrome P450	FH898882	2.88
5.	NtPMIa1g14474e2_st	Rubisco accumulation factor 1	FH386425	2.53
6.	NtPMIa1g72806e3_st	Cytochrome P450, family 704, subfamily B, polypeptide 1	FH034679	2.48
7.	NtPMIa1g32128e1_st	Chlorophyll a-b binding protein 2.1	FH437542	– 3.75
8.	NtPMIa1g121870e1_x_st	Chlorophyll a-b binding protein AB10	FH174615	– 2.38
9.	NtPMIa1g30230e1_st	Cytochrome P450, family 714, subfamily A, polypeptide 2	FH558936	– 2.36
10.	NtPMIa1g95982e1_st	Chlorophyll a/b-binding protein CP24	FH977269	– 2.06
11.	NtPMIa1g156219e1_st	Cytochrome b (mitochondrion)	FH626006	– 2.02
**Transcription factors**				
12.	NtPMIa1g48630e1_st	Transcription factor	ET758387	2.86
13.	NtPMIa1g169283e1_st	Probable WRKY transcription factor 23	FH154173	2.52
14.	NtPMIa1g31941e2_st	Heat-inducible transcription repressor	FH029374	2.49
15.	NtPMIa1g100948e1_s_st	Putative transcriptional activator DEMETER	FH999805	2.32
16.	NtPMIa1g173637e1_st	YABBY2-like transcription factor YAB2	FI080387	2.25
17.	NtPMIa1g63257e1_s_st	Nuclear transcription factor Y subunit A-10	ET739691	2.24
18.	NtPMIa1g179140e1_st	GATA transcription factor 16	ET049433	2.23
19.	NtPMIa1g14745e1_st	Probable WRKY transcription factor 4	FG172186	2.12
20.	NtPMIa1g51042e1_st	Transcription factor ILR3-like	ET854451	2.06
21.	NtPMIa1g58686e1_st	GATA transcription factor 21	FH630528	– 2.77
22.	NtPMIa1g84759e1_s_st	Putative transcription elongation factor SPT5 homolog	FH940966	– 2.40
23.	NtPMIa1g5135e1_st	NAC domain containing protein 10	FH774009	– 2.31
24.	NtPMIa1g121208e1_st	MADS-box transcription factor FBP4.	FI070259	– 2.24
25.	NtPMIa1g183221e1_st	GATA transcription factor 8-like	FH668641	– 2.03
**Receptors/signalling /Kinases**				
26.	NtPMIa1g143095e1_st	Receptor lectin kinase	FH976696	3.41
27.	NtPMIa1g120738e1_st	L-type lectin-domain containing receptor kinase IV.1	FI068742	3.17
28.	NtPMIa1g95764e1_st	Concanavalin A-like lectin protein kinase family protein	FH644056	2.48
29.	NtPMIa1g96033e1_s_st	Mannose-binding lectin superfamily protein	FH970974	2.25
30.	NtPMIa1g182212e1_x_st	Mitogen-activated protein kinase kinase kinase 5	FH974766	2.21
31.	NtPMIa1g93065e1_x_st	Putative thaumatin-like protein	FH524903	2.15
32.	NtPMIa1g26160e1_st	Serine/threonine-protein kinase-like protein	FH404353	2.12
33.	NtPMIa1g82319e1_st	Serine/threonine-protein kinase	FH087869	2.03
34.	NtPMIa1g74319e1_x_st	LRR receptor-like serine/threonine-protein kinase	ET908645	– 2.46
35.	NtPMIa1g61335e2_st	Calcium-dependent protein kinase	FH408198	– 2.44
36.	NtPMIa1g10568e1_st	Calcium-binding protein	FH215122	– 2.37
37.	NtPMIa1g122388e1_st	Mitogen-activated protein kinase kinase kinase 15	FH344502	– 2.22
**Abiotic stress responsive**				
38.	NtPMIa1g178341e1_st	Auxin-responsive family protein	FH571766	2.55
39.	NtPMIa1g77499e1_st	Cyclic Nucleotide-Regulated Ion Channel Family Protein	EH621839	2.34
40.	NtPMIa1g49198e3_st	Auxin response factor 5	ET050562	2.30
41.	NtPMIa1g24250e1_st	Auxin-responsive protein IAA6	ET790168	2.24
42.	NtPMIa1g137731e2_st	Putative chloride channel-like protein Clc-G-Like	FH201280	2.11
43.	NtPMIa1g100401e2_st	Auxin-responsive protein IAA11	FH994554	2.02
44.	NtPMIa1g36970e1_st	Auxin efflux carrier family protein	ET860447	– 2.93
45.	NtPMIa1g176858e1_s_st	Aquaporin PIP2 2 mRNA	ET782162	– 2.66
**Biotic stress responsive**				
46.	NtPMIa1g176538e1_x_st	Disease resistance protein	FI079692	2.94
47.	NtPMIa1g46074e1_st	NBS-LRR disease resistance protein homologue	FH899274	2.28
48.	NtPMIa1g49587e1_st	Pathogenesis-related thaumatin superfamily protein	ET051702	2.24
49.	NtPMIa1g57429e1_st	Plant viral-response family protein	ET737281	2.11
50.	NtPMIa1g33021e2_st	Disease resistance protein (TIR-NBS-LRR) family	ET856286	– 2.13
**Antioxidants**				
51.	NtPMIa1g22665e1_s_st	Superoxide dismutase 3, chloroplastic	FH372649	2.38
52.	NtPMIa1g10068e2_st	Glutathione S-transferase, C-terminal-like	FH199263	2.27
**Chaperon/Heat shock**				
53.	NtPMIa1g12414e1_s_st	Chaperonin 60 subunit beta 4, chloroplastic;	ET809635	3.08
54.	NtPMIa1g96842e1_x_st	Heat shock 65 kDa protein	FH979702	2.99
55.	NtPMIa1g99174e1_st	Histone chaperone ASF1B	FH990555	2.77
56.	NtPMIa1g154227e1_s_st	Heat shock 70 kDa protein	FH539518	2.44
57.	NtPMIa1g97529e1_st	Chloroplast Heat Shock Protein 70-2	FH272606	2.08
58.	NtPMIa1g742e1_s_st	Heat shock protein DnaJ with tetratricopeptide repeats	FH501867	– 2.22
**Transporters**				
59.	NtPMIa1g35785e3_st	Potassium channel	ET966045	2.84
60.	NtPMIa1g49250e1_st	Peptide transporter 3	FH083864	2.72
61.	NtPMIa1g51558e1_st	Nodulin MtN21/EamA-like transporter family protein	ET913234	2.49
62.	NtPMIa1g88526e1_st	ABC transporter family protein	FH951768	2.46
63.	NtPMIa1g77463e4_st	Plant calmodulin-binding protein-like protein	ET982476	2.38
64.	NtPMIa1g52615e1_s_st	Putative sugar transporter	EH622321	2.37
65.	NtPMIa1g66400e1_st	Auxin transport protein	FH676020	2.26
66.	NtPMIa1g107239e1_st	Nuclear Transport Factor 2 (Ntf2) Family Protein	FI022849	2.09
67.	NtPMIa1g12413e1_st	K+ transporter 5	FH098156	2.02
**Zinc fingers/leucine zipper motifs containing proteins**				
68.	NtPMIa1g48247e2_st	B-box type zinc finger protein with CCT domain	FH973157	3.45
69.	NtPMIa1g29354e1_st	Homeobox-leucine zipper protein HOX27	ET042333	2.64
70.	NtPMIa1g7510e1_st	Homeobox-leucine zipper protein HDG12	ET046033	2.38
71.	NtPMIa1g205801e2_s_st	Putative DHHC-type zinc finger protein	FI045682	2.25
72.	NtPMIa1g12465e1_st	Ring zinc finger protein-like	FH486990	2.05
73.	NtPMIa1g93322e1_st	Zinc finger (C2h2 Type) Family Protein	ET858669	2.04
74.	NtPMIa1g150572e1_st	Zinc finger and hAT dimerization domain	FI059792	– 2.39
75.	NtPMIa1g124319e1_st	B-box type zinc finger protein with CCT domain	FH540296	– 2.15
**Unknown/hypothetical/uncharacterized**				
76.	NtPMIa1g102443e1_st	Uncharacterized	FI004635	3.65
77.	NtPMIa1g18387e2_st	Hypothetical protein	FH373747	3.16
78.	NtPMIa1g16155e1_st	Uncharacterized	ET985839	3.10
79.	NtPMIa1g12003e1_st	Uncharacterized	FH258062	– 3.53
80.	NtPMIa1g18678e1_st	Uncharacterized	FG194168	– 2.56
**Transcripts significantly differentially expressed under salt (150 mM NaCl) stress condition**				
**Photosynthesis**				
1.	NtPMIa1g89206e1_st	Cytochrome P450 71A2	FH116143	6.62
2.	NtPMIa1g77065e1_s_st	Cytochrome P450	FH044298	5.98
3.	NtPMIa1g167443e1_st	Elicitor-inducible cytochrome P450 (CYP71D20)	ET820462	5.78
4.	NtPMIa1g123263e1_st	Cytochrome b561	EH620440	5.77
5.	NtPMIa1g31166e2_st	Cytochrome P450, family 71, subfamily B, polypeptide 38	FH568323	4.54
6.	NtPMIa1g87311e1_st	SufE-like protein 2, chloroplastic	FH948198	4.38
7.	NtPMIa1g119076e1_st	Cytochrome P450, family 76, subfamily C, polypeptide 4	FH505851	3.83
8.	NtPMIa1g14505e1_st	Cytochrome P450, family 76, subfamily C, polypeptide 3	FH655318	3.65
9.	NtPMIa1g32128e1_st	Chlorophyll a-b binding protein 2.1, chloroplastic	FH437542	– 5.86
10.	NtPMIa1g111798e1_st	Photosystem I light harvesting complex protein	FI040386	– 5.05
11.	NtPMIa1g48906e2_st	Photosystem I chlorophyll a/b-binding protein	FH210112	– 4.71
12.	NtPMIa1g85781e1_x_st	Light-harvesting complex II chlorophyll a/b-binding protein	FH584622	– 4.60
13.	NtPMIa1g34716e1_x_st	Photosystem II light harvesting complex protein	FH010322	– 4.51
**Transcription factors**				
14.	NtPMIa1g12272e1_s_st	WRKY transcription factor	FH228396	6.16
15.	NtPMIa1g32161e3_st	Transcriptional activator	ET898480	3.60
16.	NtPMIa1g37438e1_st	NAC domain-containing protein	ET867074	6.23
17.	NtPMIa1g84232e2_st	NAC domain-containing protein 72	FH231367	4.18
18.	NtPMIa1g61499e1_x_st	myb-like transcription factor family protein	FI051254	3.41
19.	NtPMIa1g142252e1_st	Ethylene-responsive transcription factor	FH036716	4.04
20.	NtPMIa1g48202e1_st	BZIP transcription factor bZIP77	FH071215	3.97
21.	NtPMIa1g6980e1_s_st	MYC transcription factor	FG185704	– 3.59
22.	NtPMIa1g62658e1_st	AP2 transcription factor	ET046270	– 3.46
23.	NtPMIa1g43983e1_st	Basic-leucine zipper (bZIP) transcription factor	ET042023	– 3.41
**Receptors/signalling /kinases**				
24.	NtPMIa1g183983e1_st	Serine/threonine protein kinase 2	FH678658	3.62
25.	NtPMIa1g10490e2_st	Serine/threonine kinase	FH213282	3.58
26.	NtPMIa1g182617e1_st	G-type lectin S-receptor serine/threonine-protein kinase	FH234969	3.42
27.	NtPMIa1g107535e1_st	Calcium dependent protein kinase	FH688575	– 3.54
**Abiotic stress responsive**				
28.	NtPMIa1g81893e2_s_st	Abscisic acid-responsive	FH334926	5.29
29.	NtPMIa1g25688e1_st	Hypoxia-responsive family protein	ET710946	5.14
30.	NtPMIa1g65621e1_st	Auxin-induced protein	ET676757	5.03
31.	NtPMIa1g58720e4_st	Cyclic nucleotide-gated ion channel	FH710343	4.53
32.	NtPMIa1g187181e1_st	K^+^ efflux antiporter	FH733320	4.39
33.	NtPMIa1g80304e1_st	Aquaporin	FH517170	4.11
34.	NtPMIa1g82556e4_st	Water channel protein MipK	ET846459	4.09
35.	NtPMIa1g87091e3_s_st	Early-responsive to dehydration protein	ET815593	3.69
36.	NtPMIa1g174261e1_s_st	Calmodulin	FH113769	3.69
37.	NtPMIa1g94367e1_st	Calcium binding protein	FG176020	3.64
38.	NtPMIa1g183329e1_x_st	Sodium/calcium exchanger membrane region	FI080543	3.61
39.	NtPMIa1g100023e1_s_st	Late embryogenesis abundant protein D-29	FH993271	3.51
40.	NtPMIa1g52217e3_st	K^+^ uptake permease	FH496325	3.34
41.	NtPMIa1g46641e1_st	Senescence-associated gene	ET683467	– 3.95
42.	NtPMIa1g25579e1_st	Senescence-inducible chloroplast stay-green protein	FH985884	– 3.55
**Biotic stress responsive**				
43.	NtPMIa1g50893e1_x_st	Pathogen induced protein uncharacterized	FH017913	5.30
44.	NtPMIa1g446e2_s_st	Pathogenesis-related transcriptional factor and ERF	FI004101	3.86
45.	NtPMIa1g73165e2_s_st	Pathogen induced protein	ET724700	3.80
46.	NtPMIa1g89450e2_st	Putative verticillium wilt disease resistance protein Ve2	ET690367	3.76
47.	NtPMIa1g11236e2_st	Pathogenesis-related protein Q (PR-Q)	EH618316	3.35
**Antioxidants**				
48.	NtPMIa1g2398e1_s_st	ACC oxidase	FH038492	5.83
49.	NtPMIa1g285e1_s_st	Glutathione S-transferase	FH948778	5.24
50.	NtPMIa1g107555e1_s_st	l-ascorbate oxidase	FH518217	4.16
51.	NtPMIa1g116765e2_st	Ascorbate oxidase	FI055586	3.39
52.	NtPMIa1g100148e1_x_st	Ascorbate peroxidase	FG199962	3.34
**Chaperon/heat shock**				
53.	NtPMIa1g122482e1_st	mitochondrial chaperone	EH622598	5.25
54.	NtPMIa1g80636e4_st	chaperone protein chloroplastic	ET703308	4.89
55.	NtPMIa1g48639e1_st	Heat shock 70 kDa protein	FH643753	4.16
**Transporters**				
56.	NtPMIa1g45198e1_s_st	Sugar transport protein	ET923251	5.92
57.	NtPMIa1g179293e1_st	ABC protein	FH628071	5.36
58.	NtPMIa1g36079e1_st	Amino acid transporter	FH005832	4.53
59.	NtPMIa1g58619e1_st	Sulfate transporter	FG143420	4.36
60.	NtPMIa1g12338e2_s_st	Ammonium Transporter 2	ET797276	4.02
61.	NtPMIa1g170644e1_st	High affinity K+ transporter	FH538430	3.64
62.	NtPMIa1g202149e1_st	Nitrate transporter NRT1-5	ET806108	3.38
63.	NtPMIa1g193197e1_s_st	Sugar phosphate exchanger, putative	FH747309	– 4.11
**Zinc fingers/leucine zipper motifs containing proteins**				
64.	NtPMIa1g102701e1_st	Zinc induced facilitator	FH079466	6.01
65.	NtPMIa1g95486e1_st	Zinc finger protein CONSTANS	FH975799	5.69
66.	NtPMIa1g34050e1_st	Zinc finger CCCH domain	FH744808	3.35
66.	NtPMIa1g227029e1_st	Zinc finger B-box protein	FG167857	4.06
68.	NtPMIa1g68098e1_st	DHHC-type zinc finger family protein	FG197147	3.43
69.	NtPMIa1g31503e1_s_st	b-box type zinc finger protein with CCT domain	ET051218	– 3.97
**Unknown/hypothetical/uncharacterized**				
70.	NtPMIa1g8438e1_st	Uncharacterized	FH583256	7.71
71.	NtPMIa1g110238e3_s_st	Hypothetical protein	FG133280	5.19
72.	NtPMIa1g197992e1_st	Uncharacterized transporter	ET761936	5.16
73.	NtPMIa1g191871e1_st	Uncharacterized	FH009504	– 8.35
74.	NtPMIa1g100379e1_st	Hypothetical protein	ET829011	– 6.03
**Miscellaneous**				
75.	NtPMIa1g38846e1_st	Early flowering-like protein	ET778187	4.85
76.	NtPMIa1g36370e1_st	Early nodulin-like protein	FH240198	4.79
77.	NtPMIa1g35320e3_s_st	Nodulin family protein	FI036052	4.53
78.	NtPMIa1g51558e1_st	Nodulin/EamA-like transporter family protein	ET913234	3.72

No sign indicates up-regulation, whereas “−” sign shows down-regulation. Fold-expression is significant at ANOVA *p* < 0.05.

## Discussion

Plant growth-promoting rhizobacteria (PGPR) is considered an attractive way for sustainable agriculture to cope up with biotic and abiotic stresses. However, due to difficulties in practical implication, handling in field conditions, and comparatively slow response, alternative ways are much needed^27^. The gene(s) that are differentially over-expressed in host plants in the response of plant-microbe (PGPR) interaction could be potential candidates to be explored to engineer crops for future agriculture under different stress conditions. Keeping this thought in mind, we have identified and clone genes that are differentially expressed in peanut (host plant) in the response of interaction with PGPR (*S. maltophilia*) under the N_2_ starvation conditions (Fig. 1)*.* These differentially expressed genes could be utilized to improve crop productivity in nitrogen-deficient and salt-affected areas. In a previous study, we reported that *S. maltophilia* BJ01 modulates the physiology of peanut plants to protect them under nitrogen deficiency and salt stress conditions^19,20^. In this study, first and foremost we identified the differentially expressed gene due to interaction of *S. maltophilia* BJ01 under nitrogen starvation condition.

PCR-based cDNA subtraction, commonly known as suppression subtractive hybridization (SSH), is a powerful method for selectively amplification of differentially expressed target cDNA and at the same time, non-targeted DNA amplification is suppressed^28^. The SSH result showed that about 60% of differentially expressed genes were of unknown/uncharacterized function. Expression of a large number of uncharacterized or hypothetical genes after interaction with PGPR under the N_2_ deficit condition provides a molecular insight of changes that occurs during the interaction of *S. maltophilia* and *A. hypogaea*. In contrast, the interaction between *Cicer arietinum* and *Ascochyta rabiei* resulted in 7% genes of unknown function^29^, whereas the interaction between *Vitis pseudoreticulata* with *Uncinula necator* leads to the differential expression of 24% uncharacterized genes^30^. These N_2_ starvation-responsive genes were further validated by qRT-PCR, and the expression profiling of these uncharacterized SSH clones showed that these genes were up-regulated during plant interaction with *S. maltophilia* (Fig. S1)*.* An N_2_ deficiency and PGPR interaction responsive clone SM409 (later on named the *AhCytb6* gene, which shows the similarity with PSII related gene *cytb6*), had higher expression (about 4-fold) among studied clones in a transcript profiling and was selected to characterize further in a model plant tobacco. Fataftah et al. showed that 1938 genes were differentially expressed in barley leaves after 20 days of nitrogen starvation; when plants were resupplied with nitrogen, 62% of genes that were down-regulated were up-regulated and out of these genes, most of the genes belong to photosynthesis^31^. The RNAseq data of Yang et al. showed that the *Cytb*/f complex is upregulated in leaves of low nitrogen level tolerance verity of sugarcane; both studies indicate the involvement of *Cytb6* gene in nitrogen deficit condition^32^. Thus, this study also supports the major role of photosynthetic related genes in the case of nitrogen starvation. In contrast, PSII related genes were downregulated in durum wheat under nitrogen starvation conditions^33^. Thus, differential expression of gene under N_2_ starvation is due to interaction with the *S. maltophilia* and helps plants to cope up with the nitrogen starve condition. The genome organization study confirmed that the *AhCytb6* gene is intronless, and *in-silico* analysis revealed that the gene encodes for a transmembrane protein consisting of helix and coil motifs that is highly stable (Figs. S3–S4).

All raised transgenic lines were checked for the confirmation of transgene, and out of 17 transgenic lines (Fig. 1), we selected five lines showing single transgene integration with the high expression for further analysis under stress conditions. Overexpression of *AhCytb6* improved seed germination and health of the growing seedlings under N_2_ starvation and salt stress conditions where WT and VC failed to do so (Figs. 2, 3). Transgenic seedlings grown in stress conditions exhibited higher shoot length, root length, fresh weight, and dry weight in comparison to WT and VC (Fig. 3). The enhanced germination and growth of the transgenic plants showed that the *AhCytb6* gene increases the tolerance against N_2_ starvation and salt stress by restoring the photosynthetic machinery and equilibrating C:N ratio, which is important during the reproductive and growth period of the plant. In another study, Qiao et al. showed that Cytochrome b561 was differentially expressed and up-regulated in pigeon pea after interaction with arbuscular mycorrhizal fungi under drought, which supports the role of the *cytb6* gene in legumes in the symbiotic relationship under abiotic stress condition^34^. On the other hand, Dyda et al. showed that cytochrome b559 was down-regulated in triticale after infection with pathogenic fungus *Microdochium nivale,* which showed that gene *cytb* has a crucial role in plant immunity^35^. Joaquín-Ramos et al. showed that *CYTb6f* was significantly up-regulated in *Amaranthus cruentu* under salt stress (300 mM) which supports the role of *cytb6* under salt stress^36^. Constitutive expression of rice microRNA528 also showed enhanced growth, elevated biomass, and tolerance to salinity stress and N_2_ starvation in the transgenic plants^37^.

The physiological status of the plant determines growth and survival during harsh environmental conditions. Abiotic stress damages the plant cell membrane, integrity of the cell membrane is essential for the stress tolerance of the plant. Results of EL and MSI showed that all transgenic plants overexpressing the *AhCytb6* gene had less cell membrane injury compared to WT and VC plants (Fig. 4). Thus, increased membrane stability and a low level of electrolyte leakage help the plant to maintain the cell homeostasis under stress conditions; similar results were obtained by Ben-Romdhane et al.^38^. Proline is an essential osmolyte and molecular chaperon, which helps the plant to maintain the cytosolic redox status and ROS scavenging as well as helping the plant under stress conditions^39,40^. The transgenic plant overexpressing the *AhCytb6* gene enhanced the proline production in transgenic plants and enabled plants to mitigate the stress conditions at the cellular level.

The ROS metabolism in the cell is regulated by redox mechanisms with the help of antioxidants, and it maintains the stable dynamic equilibrium in normal physiological conditions^41^. Under stress conditions, this balance is disrupted and creates oxidative stress, which can cause damage to nucleic acids, proteins, and lipids^42,43^. When living cells face stress, they generate free radicles like superoxide, hydrogen peroxide, and cell membranes (which are made up of fatty acids) prone to oxidation. These free radicles cause peroxidation of the cell membrane and generate malondialdehydes (MDA); thus, these parameters are used for the biochemical markers to measure the stress levels^44^. Abiotic stress causes disturbance in PSII, which causes the generation of a high amount of ROS; here overexpression of *AhCytb6* may provide stability to ETC and cause a reduction in ROS in transgenic lines. The WT and VC plants accumulate more MDA, H_2_O_2,_ and O_2_^-^ in comparison to transgenic plants (Fig. 4). Thus, results confirmed the role of *AhCytb6* in ROS scavenging in plants and stress tolerance of N_2_ starvation and salt stress conditions. Recently, Yang et al. also showed that transgenic tobacco lines overexpressing chloroplast targeting and heme-binding genes *AhFC1* and *AhHEMA1* had less accumulation of MDA, H_2_O_2_ content in comparison to the wild-type under 200 mM salt stress conditions^45^. This finding also supports the role of chloroplast targeting and cytochrome-related genes in plant defense mechanisms other than photosynthesis.

Salt stress can cause damage to the chlorophyll pigment-protein complex and degrades the enzyme chlorophyllase, and nitrogen starvation causes chlorosis in leaves^46,47^. Chlorophyll contents observed under salt and N_2_ deficit condition showed that the transgenic plants were able to retain more chlorophyll contents and carotenoids in comparison to the WT and VC counterparts (Fig. 5). A higher concentration of carotenoid content in transgenic lines is an indicator of better photosynthetic efficiency as well as reduced oxidative stress in stressed conditions because carotenoid also plays a protective role against ROS^48^. It is quite evident that *AhCytb6* protects plants from the loss of chlorophyll and helps the information of vital pigments via improving performance in photosynthesis. Under low nitrogen, plants reduce their photosynthesis to reduce energy loss; we found that the net photosynthesis rate was significantly higher in the transgenic plants in N_2_ starvation in comparison to WT and VC. Electron transport is very much affected during photosynthesis by high salt concentration and/or nitrogen starvation, which deteriorates the photosynthetic performance of plants^23,49^. In our study, transgenic lines overexpressing the *Ahcytb6* gene has a higher net photosynthesis rate, stomatal conductance, and transpiration rate than WT and VC show that the *AhCytb6* gene plays a key factor in PSII and enhances photosynthesis efficiency and yield of the plant under stress (Fig. 6A and Fig. S6). Similar activity of the *OsPGK2-P* gene was also reported in transgenic tobacco under salt stress^50^.

Microarray analysis of the plant overexpressing *the AhCytb6* gene showed differential expression of the gene compared to the WT plant in similar stress conditions. Our results showed that the ectopic expression of *AhCytb6* influenced the expression of genes belonging to metabolism (28%), transporters (9%), photosynthesis (7%), abiotic stress-responses (7%), receptor/kinase/signaling (7%), uncharacterized (6%), transcription factors (5%), biotic stress (3%), antioxidant (1%), and chaperon/heat shock protein (1%). Besides this, miscellaneous (21%) and housekeeping (3%) were also differentially expressed under salt stress. Results coincided with the study of Passricha et al.^51^, where transporters, kinases, and genes related to abiotic stress were also differentially expressed in transgenic rice overexpressing the *PsLecRLK* gene under salt stress. Under N_2_ starvation conditions metabolism (19%), transporter (4%), photosynthesis (3%), abiotic stress-responsive (5%), receptor/kinase/signaling (8%), uncharacterized (6%), transcription factors (4%), biotic stress (2%), and chaperon/heat shock protein (3%) along with miscellaneous (34%) and housekeeping (8%) were also differentially expressed.

**Figure 8:**
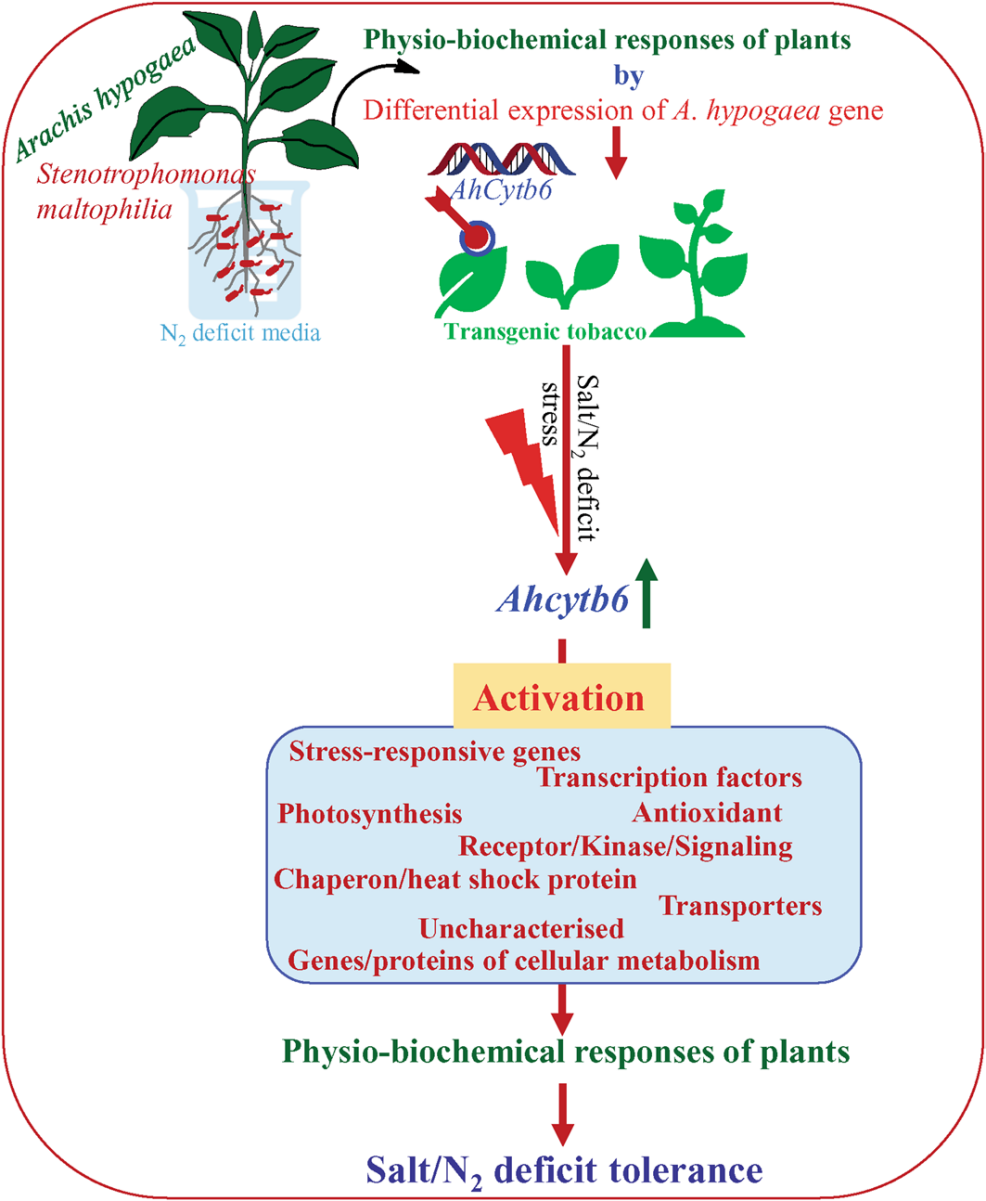
A hypothetical schematic model explaining the probable role of the *AhCytb6* abiotic stress tolerance system.

Transgenic tobacco overexpressing wheat microRNA *TaMIR444a* led to the differential expression of 1733 genes in compression of WT under N_2_ starvation. These genes belonged to unknown, transcription, transportation, abiotic and biotic stress, signaling, metabolism, and a miscellaneous category^52^. Microarray data show that overexpression of the *AhCytb6* gene affects the plant response in nitrogen starvation and salt stress at the molecular level, these changes cumulatively support a plant under stress condition, and transgenic lines perform better than the wild-type counterparts do. Overexpression of abiotic and biotic stress-related genes and heat shock proteins show that this gene can play important role in priming plant immunity under biotic and abiotic stresses. In a study by Luo et al., nitrogen availability is directly proportional to the differential overexpression of photosynthesis-related genes^53^. In contrast to this, due to the overexpression of the *Cytb6* gene, 3% of photosynthetic genes were differentially expressed under N_2_ starvation, showing the role of this gene in N_2_ assimilation, which follows a recent study of Iqbal et al.^54^. Overexpression of transcription factors like WRKY, GATA, YAB2 under N_2_ starvation and WRKY, NAC, MYB, under salt stress show that the *Cytb6* gene plays a major role in C–N metabolism and in salt stress that starts at the transcription level. Rubisco, which is an indicator for total N_2_ level in plants and leaves, also up-regulates in transgenic plants under salt and N_2_ starvation showing the balancing role of Cytb6 in the C–N cycle in stress conditions. Similar results were observed by Xin et al.^55^. Based on the above results, we hypothesized a model that summarized the probable role of *AhCytb6* in plant-microbe interaction and abiotic stress tolerance (Fig. 8).

## Conclusion

In this study, we reported the changes at the molecular level in the host plant after plant-microbe interaction. *AhCytb6* is a photosynthetic gene differentially expressed after interaction between *Arachis hypogaea–Stenotrophomonas maltophilia* under N_2_ stress. This gene plays a significant role in plant-microbe interaction, and its role is functionally validated by ectopic overexpression in transgenic tobacco plants. Morphology, physiology, biochemical, and genetic parameters were analyzed under N_2_ starvation and salt stress conditions and compared to their wild-type counterparts. We observed that transgenic plants perform better under stress conditions than WT and VC. Overexpression of this gene enhanced endurance against N_2_ starvation and salt stress. Microarray analysis of transgenic plants showed that this gene also affects the transcript expression of different stress-responsive genes and transcription factors. Overall results reveal roles of *AhCytb6* in stress tolerance under N_2_ deficit and salt stress other than photosynthesis. This gene could be further explored for the development of genetically modified crops for sustainable agriculture under N_2_ deficits and/or salt-affected areas.

## Materials and methods

### PGPR treatment to the peanut plant

Peanut (*Arachis hypogaea*) seeds (cultivar GG-20) were obtained from the Junagadh Agricultural University, Junagadh (Gujarat), which also have the voucher specimen for the identification of plants. Procure peanut seeds were surface sterilized, germinated, and transferred to previously optimized hydroponics conditions^19,56^. Briefly, seven days old seedlings were transferred 300 mL ½ MS media supplemented without nitrogenous component. Plants were treated with plant growth-promoting rhizobacterium *S. maltophilia* BJ01, which has proven ability to provide plant tolerance under N_2_ deficit conditions^19^. Plants were grown under the N_2_ starvation condition with (T) or without bacterial inoculation (C) for 21 days. Plant leaves were collected after 21 days and stored at − 80 ℃ for further experiments.

### Suppression subtractive hybridization (SSH) and dissemination of differentially expressed gene(s)

Total RNA was isolated from control and treated plants by guanidine isothiocyanate (GITC) method, and mRNA was purified using Poly AT tract mRNA isolation kit following the manufacturer's instruction (Promega, USA). Total 2 µg mRNA was used for single stranded cDNA synthesis using 1 mM primer (5′-TTT TGT ACA AGC TT_30_ N_1_N-3′ containing *Rsa* I restriction sites; GTAC), 1 mM deoxynucleotides (dNTPs), and 20 units of avian myeloblastosis virus (AMV) reverse transcriptase. Immediately after completion of single stranded cDNA, proceed for the double stranded cDNA synthesis at 16 ℃ in thermal cycler using 0.2 mM dNTPs mix, a second strand enzyme cocktail (containing DNA polymerase I, RNase H and DNA ligase) and 6 unit of T4 DNA polymerase^57^. The cDNA, synthesized from the treated plant was considered as a ‘tester’ while the cDNA of the control plant was used as a ‘driver.’

Suppression subtractive hybridization (SSH) was performed with a PCR-Select cDNA Subtraction Kit according to the user manual (Clontech, USA). In brief, the blunt end was created in driver and tester double-stranded cDNAs by *Rsa I* restriction digestion. The tester cDNA was purified and subdivided into two sets, and each was ligated with different adaptors at 16 ℃ for 12 h. However, driver cDNAs were not ligated with any adapters. Hybridization of tester and driver cDNA was carried out in two steps; in the first step, digested driver cDNA was added to individual adapter-ligated tester cDNA, denatured at 98 ℃ for 90 s and allowed for hybridization at 68 ℃ for 8 h. In the second step, both hybridized products were mixed, and the fresh denatured driver cDNA was added and allowed for hybridization again. Differentially expressed cDNAs were exponentially amplified using adapter-specific primers, cloned in *pGEM-T* easy vector (Promega, USA) and transformed into *E. coli* DH5α cells^57^. Positive clones were selected, confirmed, sequenced (at M/s Macrogen Inc., South Korea), and analyzed by bioinformatics tool.

### Selection and transcript profiling of differentially expressed genes

Differentially expressed genes obtained by SSH were categorized, and representative primer sets were designed for each category (Table S1). Total RNA was isolated (from control and treated plants), and cDNA was synthesized from 5 µg of total RNA using the ImProm‐II Reverse Transcription System (Promega, USA). Quantitative real-time (qRT)-PCR reaction was performed with Power SYBR Green PCR Master Mix (Invitrogen, USA) in a Bio-Rad CFX96 detection system (Bio-Rad, USA). The specificity of qRT-PCR was determined by melt curve analysis followed by 1% agarose gel electrophoresis. The relative fold expression of genes was calculated by the 2^−ΔΔCT^ method^58^, while actin was used as the housekeeping gene.

A clone SM409 (538 bp), which was classified in the unknown category (showing resemblance with unknown mRNA from NCBI database), and showed about 4-fold up-regulation in the treated plant (compared to the control under stress conditions), was selected for the further study.

### Cloning of gene and bioinformatics analysis

Deferentially expressed gene SM409 was made full by rapid amplification of cDNA ends, cloned in *pGEM-T* easy vector (Promega, USA), transformed to *E. coli* DH5α cells, and sequenced (at M/s Macrogen Inc., South Korea). The contiguous sequences obtained through RACE (3′RACE and 5′RACE) were assembled to obtain the full-length gene sequence. The gene-specific primer (Table S2) was designed, and a full-length gene was amplified from the cDNA of *A. hypogaea* using proof-read (*Pfu*) polymerase, cloned in *pGEM-T* easy vector and sequenced (M/s Macrogen Inc., South Korea). The sequence was analyzed using different bioinformatics tools available at the ExPASY portal. Based on different bioinformatics analyses, the SM409 clone sequence was named as the *AhCytb6* gene.

### Genetic transformation of tobacco and generation of transgenic plants for the functional analysis of *AhCytb6* gene

The complete coding region of the *AhCytb6* gene was amplified (Table S2) and cloned into the pRT100 vector down-stream to the 35S promoter. Recombinant pRT100 (pRT100:*AhCytb6*) was digested with enzyme *PstI*, expression cassette (*35S:AhCytb6:35S-ter*) was obtained and cloned in pCAMBIA2301 vector. The resultant plant expression vector pCAMBIA2301:*35S:AhCytb6* was mobilized into *Agrobacterium tumefaciens* strain EHA105 for the genetic transformation. *Agrobacterium*-mediated genetic transformation of *Nicotiana tabacum cv.* Petit Havana with the *AhCytb6* gene was done using the leaf disc transformation method^59^. After genetic transformation, leaf disc was regenerated as per standard tissue culture protocol, putative transgenic lines (T_0_) were screened on kanamycin (50 mg L^−1^) for the selection, positive plants were transferred in the greenhouse under controlled condition, and matured seeds (T_0_) were collected^60,61^.

### Analysis of transgenic lines under different abiotic stress condition

Transgenic seeds were germinated on kanamycin (50 mg L^−1^), and T_1_ transgenic lines were obtained. Transgene integration was confirmed by PCR amplification of *uidA* (GUS) and *AhCytb6* gene (Table S2); however, transgene event (copy number) was checked by southern blot analysis. Transgenic lines were subjected for histochemical GUS analysis, and five lines (L1, L4, L5, L9, and L10) were selected, and overexpression of the *AhCytb6* gene was analyzed by semi-quantitative reverse transcriptase PCR (Table S2). The selected transgenic lines were studied for morphological, biochemical and physicochemical analyses, and compared with wild-type (WT: untransformed tobacco plant) and vector control (VC: transgenic lines transformed with pCAMBIA2301 vector) plants under different abiotic stress treatments. Germination efficiency (% germination) of transgenic lines were studied under N_2_ starvation and NaCl (150 mM) stress conditions.

For the morphological study, seeds (transgenic lines, WT and VC) were germinated on MS media supplemented with kanamycin (50 mg L^−1^), and 3 days old equal size seedlings were transferred to different petri-plates (containing MS media) and subjected to N_2_ starvation and NaCl (150 mM) stress conditions for 21 days (8 h dark/16 h light cycle at 25 °C). Growth parameters were measured and documented^62^. For stress treatments, 21 days-old seedlings (grown on MS media supplemented with kanamycin) were transferred to hydroponics (containing ½ strength of MS media without N_2_ source) and grown further 21 days under N_2_ starvation conditions. In a parallel set of experiment, forty-two days old plants grown under normal conditions (1/2 MS media without any stress) were subjected to NaCl (150 mM) stress conditions for 24 h. Plants (transgenic lines: L1, L4, L5, L9, and L10; WT and VC) grown under control (without any stress) or different stress conditions (N_2_ deficit and NaCl stress) were harvested and studied for different morphology, biochemical and physiological analyses.

Leaves disc (~ 8 mm) of plants (42 days) grown (as above) under control (unstressed) conditions were subjected to different stresses (N_2_ deficit and NaCl stress) conditions for 7-days. Leaf senescence was documented, whereas chlorophyll and carotenoids were measured^56^. Comparative physio-biochemical analyses, including, electrolyte leakage, membrane stability index, proline, lipid peroxidation (MDA) content and H_2_O_2_ content were performed for all harvested plants^63-68^. The *in vivo* localization of hydrogen peroxide (H_2_O_2_) and supero xide radicals (O_2_^−^) was done by histochemical staining^69^. Photosynthesis parameters, including net assimilation rate, stomatal conduct ance, and transpiration rate, were measured by portable photosynthesis (LI6400XT, LI-COR Biosciences, USA) system^62^.

### Expression profiling of transgenic plants by microarray

A transgenic plant that performed better compared to other lines was selected for the differential transcript expression profiling^62,70^. Forty-two days old plant grown under N_2_ starvation (21 days) stress condition and a plant (42-days old) subjected to NaCl (150 mM for 24 h) stress were used for microarray analysis along with corresponding control plants. Total RNA was extracted from treated and WT (unstressed) plants and converted to first strand cDNA followed by second strand cDNA synthesis. *In vitro* transcription was performed and a cRNA was synthesized and finally converted to single-stranded cDNA. Single-stranded cDNA was fragmented, labeled and hybridized with a whole gene tobacco array, which was comprised of 272,410 gene probes. Hybridization was performed at 42 °C for 16 h, according to the user manual (Affymetrix, USA). After hybridization, the array chip was washed and stained in the fluidics module (Fluidics Station 450, Affymetrix, USA), scanned (Scanner 3000 7G, Affymetrix, USA), and analyzed using expression console (version 1.1) and transcriptome analysis console (version 3.0) software (Affymetrix, USA).

### Statistical analysis

All experiments were performed in triplicates, and each set of experiments contained five plants (except microarray, which was performed in duplicate). Statistical analysis was performed by GraphPad Prism software. All data were subjected to analysis of variance (ANOVA) followed by Dunnett test to compare all column vs WT in each condition. Values are expressed as the mean ± SE, and p value < 0.05 is considered as statistically significant.

## Supplementary information

Supplementary Information.

## Data Availability

All datasets presented in this study are included in the article and supplementary data. Microarray data are available in the ArrayExpress database (http://www.ebi.ac.uk/arrayexpress) under accession number E-MTAB-9307.
