# Supplementary material for: Overexpression of differentially expressed AhCytb6 gene during plant-microbe interaction improves tolerance to N2 deficit and salt stress in transgenic tobacco
Source: Sci Rep. 2021 Jun 28;11:13435. doi: 10.1038/s41598-021-92424-4 (PMC8239016; doi:10.1038/s41598-021-92424-4)
Supplement: Supplementary file 1 — Supplementary Information. [file 41598_2021_92424_MOESM1_ESM.pdf]

Overexpression of differentially expressed *AhCytb6* gene during plant-microbe interaction improves tolerance to N_2 deficit and salt stress in transgenic tobacco

Ankita Alexander[1,2], Vijay K Singh[1#] and Avinash Mishra[1,2]*

[1] Division of Applied Phycology and Biotechnology, CSIR- Central Salt and Marine Chemicals Research Institute, G. B. Marg, Bhavnagar- 364002, Gujarat, India

[2] Academy of Scientific and Innovative Research (AcSIR), CSIR, Ghaziabad, India

[#]Current Address: Department of Microbiology, Harvard Medical School Boston, Massachusetts, USA and Department of Surgery, Massachusetts General Hospital, Boston, Massachusetts, USA

Ankita Alexander:	alexander.ankita@yahoo.in
Vijay K Singh:	vsvijaykr@gmail.com; vijay.singh@mgh.harvard.edu
Avinash Mishra:	avinash@csmcri.res.in; avinashmishra11@rediffmail.com

*Corresponding authors:

e-mails: avinash@csmcri.res.in; avinashmishra11@rediffmail.com (AM)

Tel: +91-278-2567760 Ext. 6260; Fax: +91-278-2567562

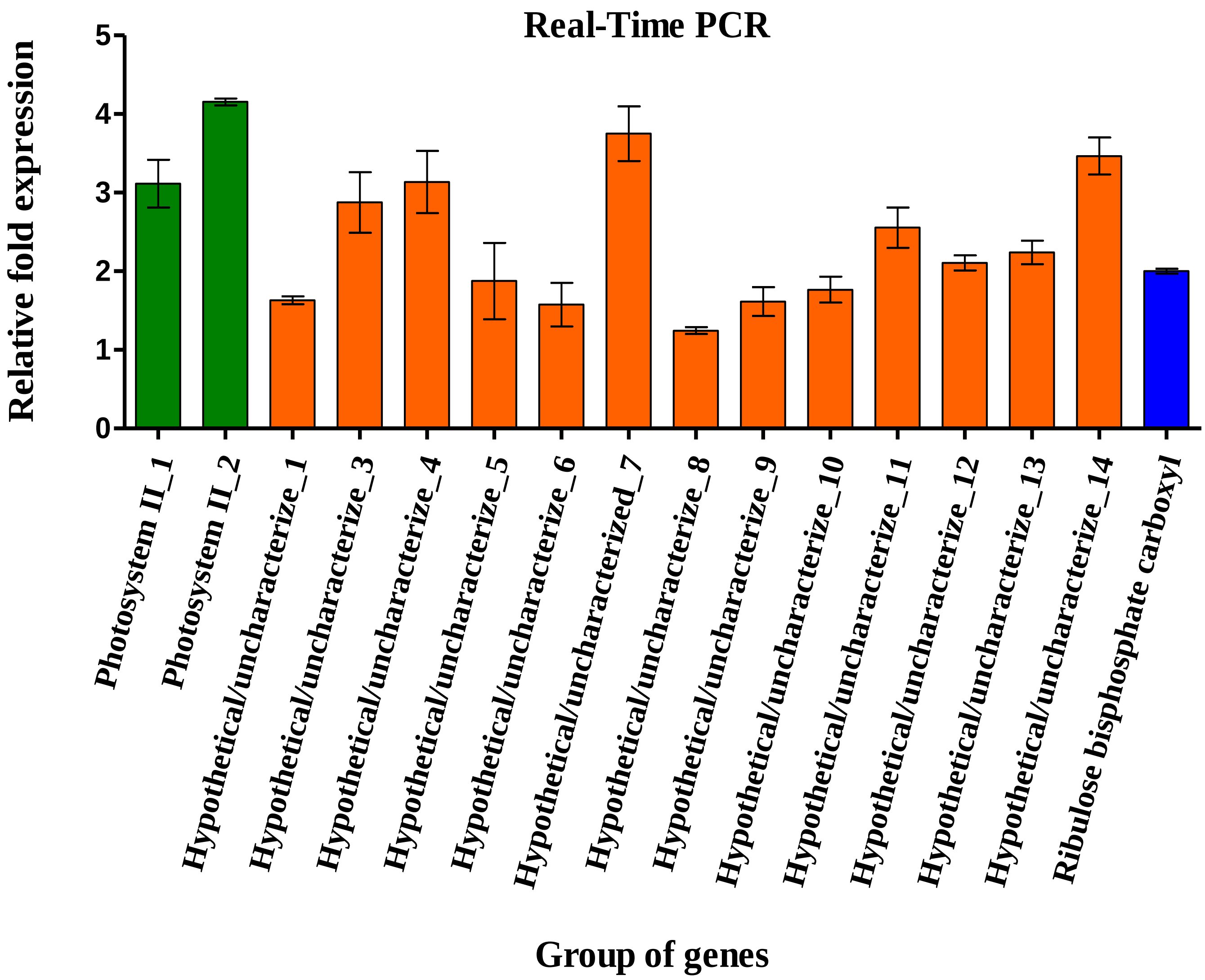

Figure S1: Transcript profiling of selected suppression subtractive hybridized clones. Representative genes from selected category of differentially expressed genes (SSH clones; Fig. 1A) showed up-regulation in PGPR treated peanut plant

***Ahcytb6* gene (MT395343)**

Up-stream region

AGTTCCTTTTGGTTTACCTATCTCAATAAAGTCT

ORF

ATGATTGGTTCGAAGAACGTCTTGAGATTCAGGCGATTGCCGATGATATTAACTAGTAAATATGTCCCTCCTCATGTCAACATATTCTATTGTTTA
GGAGGAATTACACTTACTTGCTTTTTAGTCCAAGTAGCAACGGGGTTTGCTATGACCTTTTATTATCGTCCGACCGTTACTGAGGCTTTTGCTTCG
GTTCAATATATAATGACTGAGGCTAACTTTGGTTGGTTAATCCGATCTGTTCATCGATGGTCGGCAAGTATGATGGTTTTAATGATGATCCTGCAC
GTATTTCGTGTGTATCTTACCGGCGGTTTTAAAAAACCTCGTGAATTGACGTGGGTTACGGGCGTTGTTTTGGCTGTATTGACCGCATCTTTTGGT
GTAACTGGTTATTCCTTACCTTGGGACCAAATTGGTTATTGGGCAGTCAAAATTGTAACAGGAGTACCCGAAGCTATTCCCGGAATAGGATCGTCT
GTCGTGGAATTATTAAGGGGAAGTGCTAGTGTAGGACAATCTACCTTGACTCGTTTTTATAGTTTACATACTTTTGTATTACCTCTTCTTACTGCT
GTATTTATGTTAATGCACTTTCTAATGATACGTAAGCAAGGCATCTCCGGTCCTTTATAG

down-stream region

AGAATATGGATCCTAGATATTTCTAATCAATCATTTTTTATTTTGGGGAGGAACAAAAGTATTTCATTGCTACAAATATGGATTATTAAAAAAAAT
AAGACATGTATTTGGATATTTCCCTGCAACTTAAGACTTAACAAAATTAGCGTCTTATTTTTTTATTTGACATACACGAATAGTTGATAGTTGAGG
GGGATTCTCCGAAGAAAAAACGGATTATGGGAGTAACAAAAAAACCTGACTTGAATGATCCTGTATTAAGAGCGAAATTGGCTAAAGGAATGGGTC
ATAATTATTATGGAGAACCCGCATGGCCAAACGATCTTTTATATATTTTTCCCGTAGTTATTCTAGGTACTATTGCTTGTAACGTAGGTTTAGCAG
TTTTAGAACCATCAATGATTGGGGAACCCGCGGATCCATTTGCAACTCCTTTGGAAATATTGCCGGAATGGTATTTCTTTCCTGTATTTCAAATAC
TTCGTACAGTGCCCAATAAGTTATTGGGCGTTCTTTTAATGGTTTCGGTACCCGCGGGATTATTAACAGTACCTTTTTGGAGAATGTTAATAAATT
CCAAAACCCATTCCTTTAGCCAAGCTTGAGCTCGAGTCCTC

Ahcytb6 protein

MIGSKNVLRFRRLPMILTSKYVPPHVNIFYCLGGITLTCFLVQVATGFAMTFYYRPTVTEAFASVQYIMTEANFGWLIRSVHRWSASMMVLMMILH
VFRVYLTGGFKKPRELTWVTGVVLAVLTASFGVTGYSLPWDQIGYWAVKIVTGVPEAIPGIGSSVVELLRGSASVGQSTLTRFYSLHTFVLPLLTA
VFMLMHFLMIRKQGISGPL

Figure S2: Graphical representation of *Ahcytb6* gene. The *Ahcytb6* gene was 1287 base pair long and comprised of 34 bp 5′-untranslated leader sequences (5′-UTR: 1–34 bp), 636 bp of an open reading frame (ORF: 35–670 bp) and 617 bp of a 3′-UTR (671–1287 bp). The 636-bp ORF encodes a peptide of 211

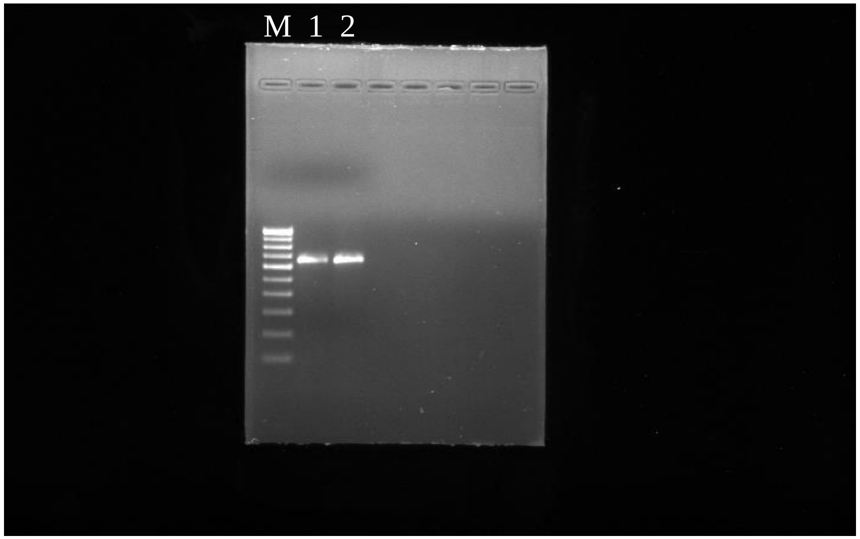

Figure S3: Genome organization

(Original and unprocessed gel-blot)

Lane M: Marker 100bp,

Lane 1: PCR amplification of *Ahcytb6* gene from genomic DNA

Lane 2: PCR amplification of *Ahcytb6* gene from cDNA

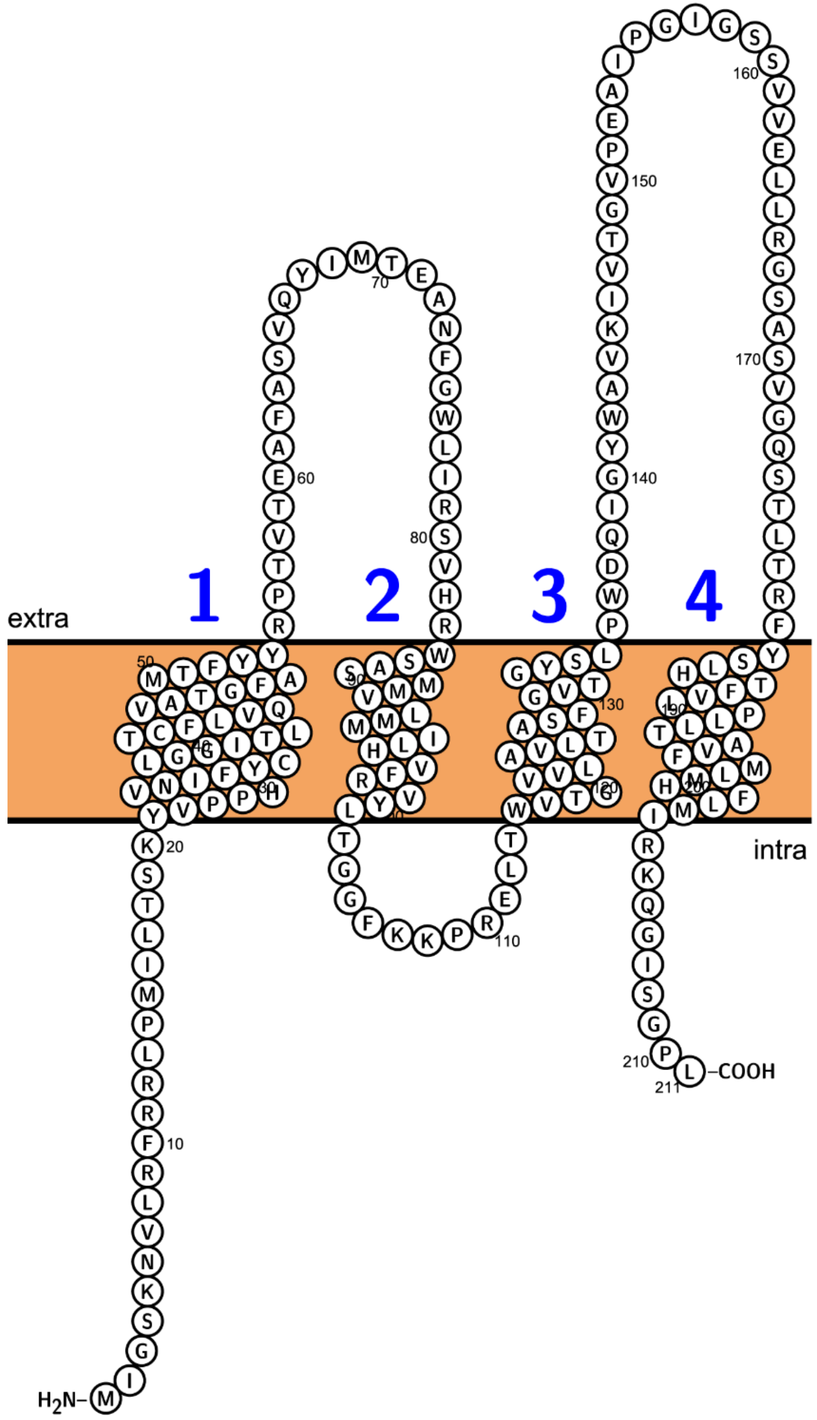

Figure S4: *In silico* localization analysis of *Ah*Cytb6 peptide. The *in silico* analysis predicted that *Ah*Cytb6 peptide contained four trans-membrane domains and localized in the plasma membrane

Structure was predicted using on-line tool available at: https://wlab.ethz.ch/protter/start/

Reference:

Omasits, U., Ahrens, C. H., Müller, S., Wollscheid, B. Protter: interactive protein feature visualization and integration with experimental proteomic data. *Bioinformatics*. **15,** 30(6), 884-886 (2014). doi: 10.1093/bioinformatics/btt607.

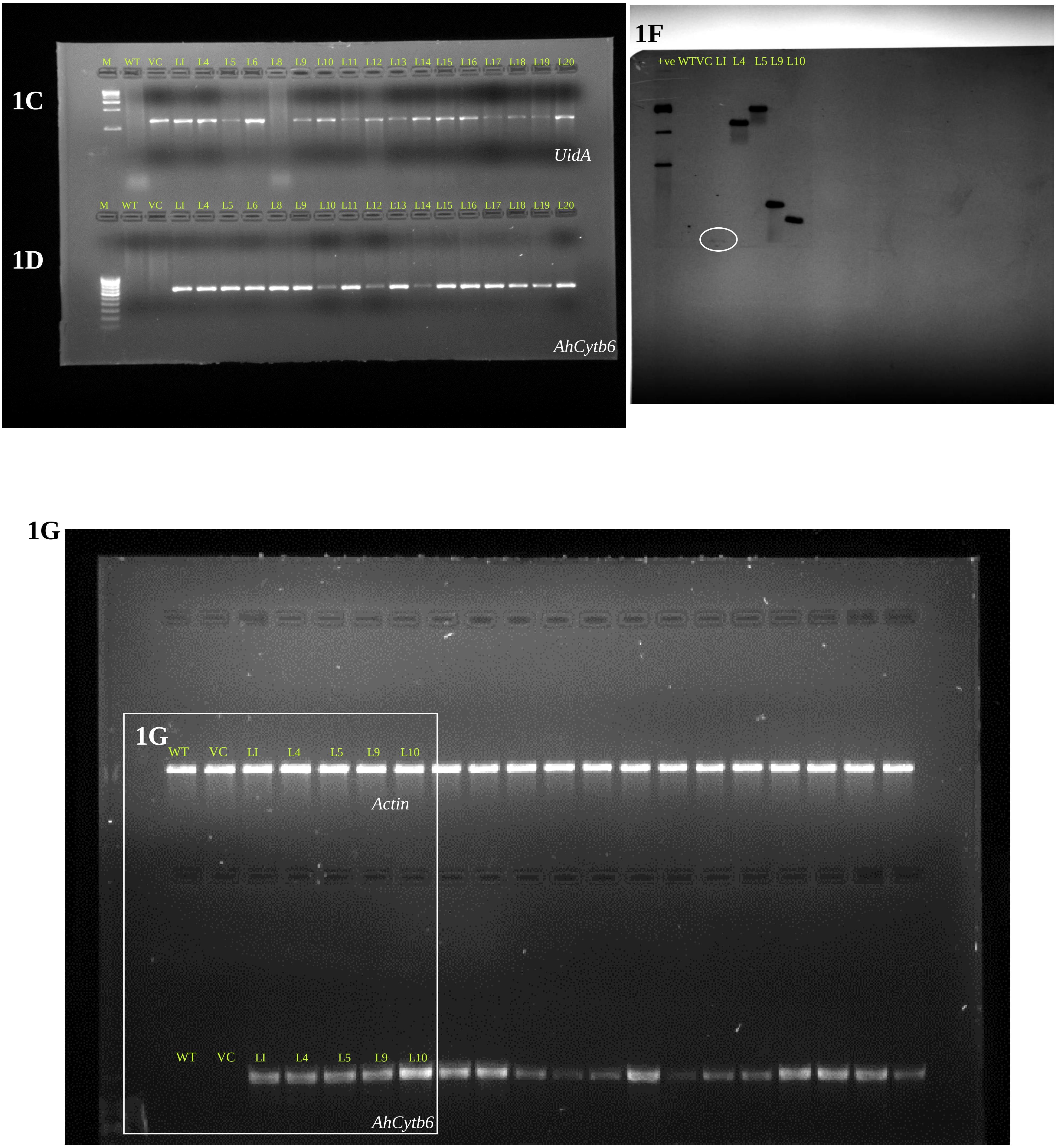

Figure S5: Original and uncropped gel-blots

Original and uncropped gel-blots of Fig. 1C, 1D, 1F and 1G

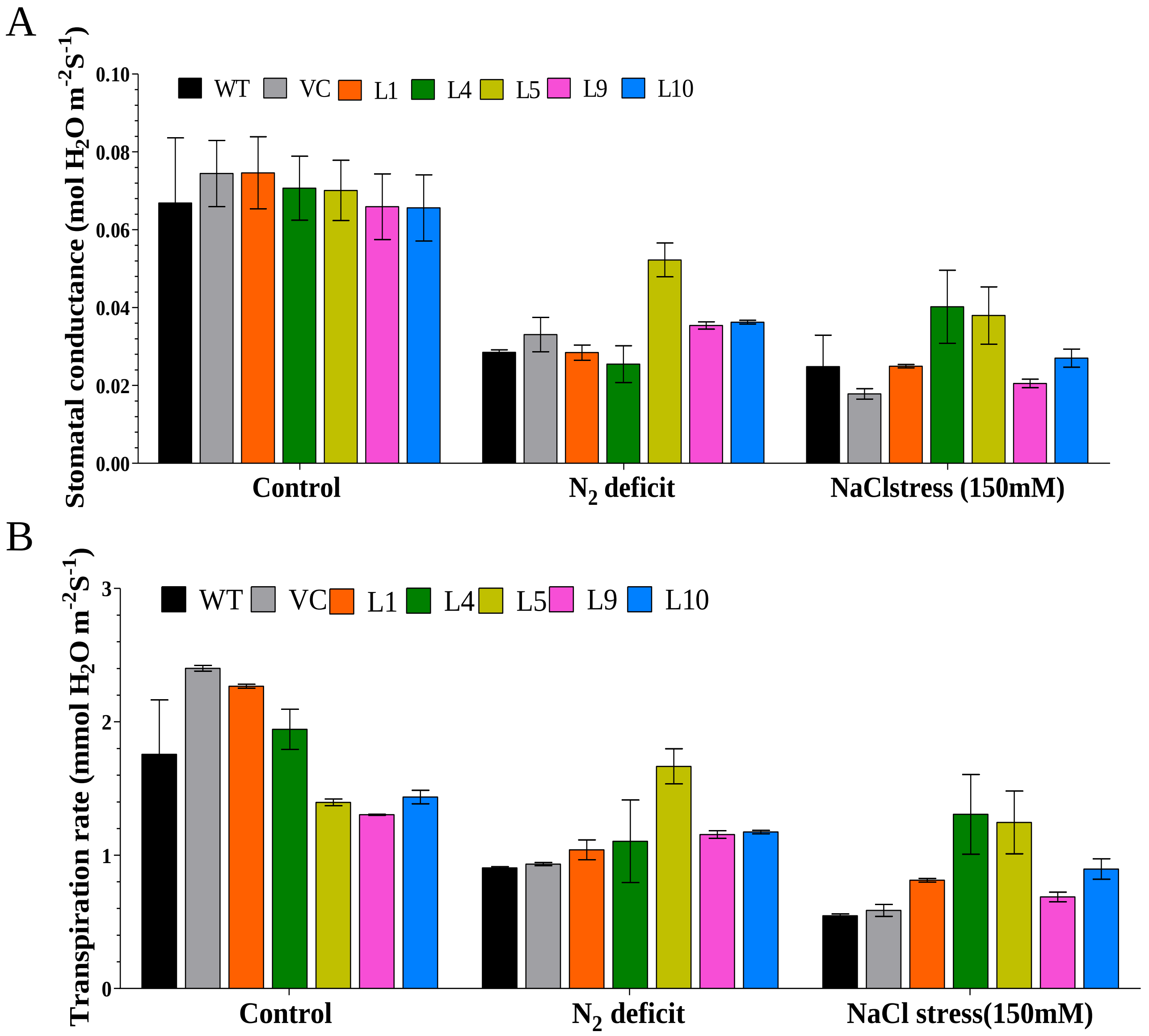

Figure S6: Stomatal conductance (A) and transpiration rate (B) in transgenic lines and control plants under control, nitrogen deficit and salt stress conditions.

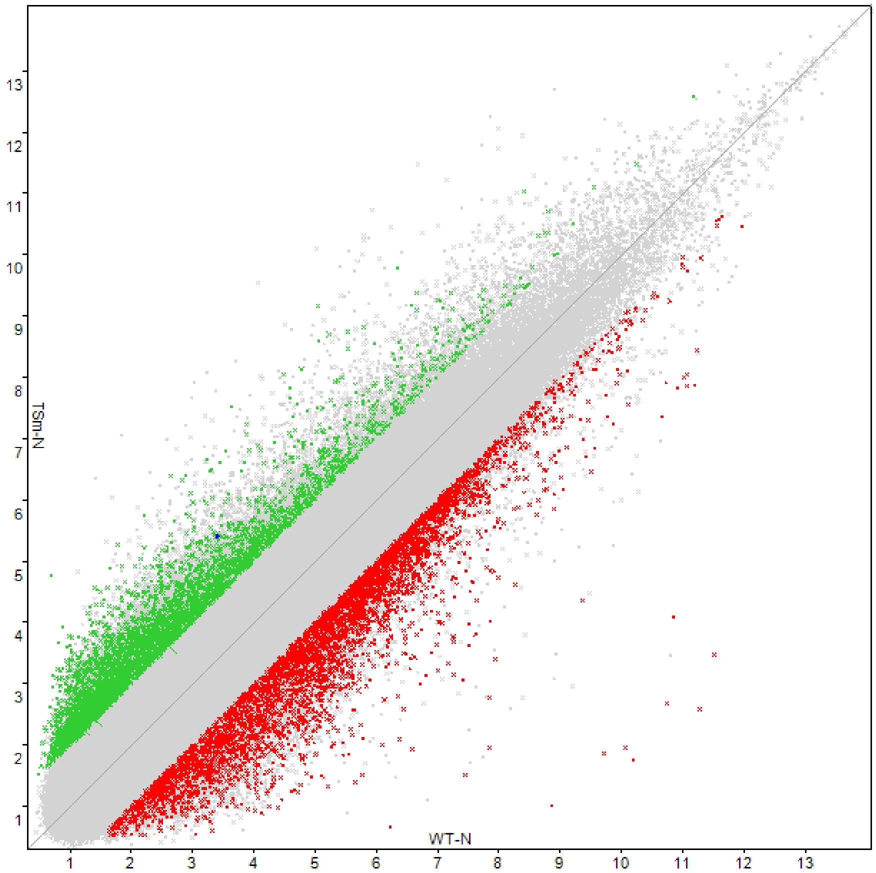

N_2 deficit condition

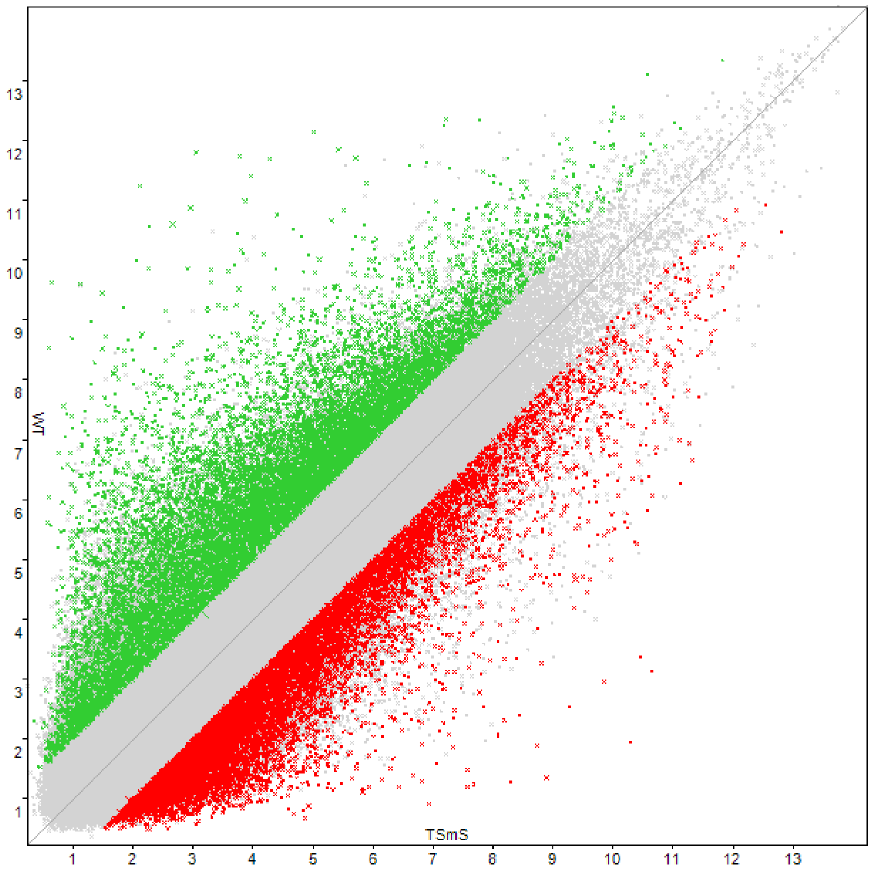

Salt stress condition

Figure S7: Scattered plot obtained from microarray analysis of *AhCytb6* transgenic tobacco plant showing differentially expressed genes under nitrogen and salt stress conditions compared to WT plant. Green and Red color dots show up- and down-regulated (2 to -2 fold) transcripts, respectively

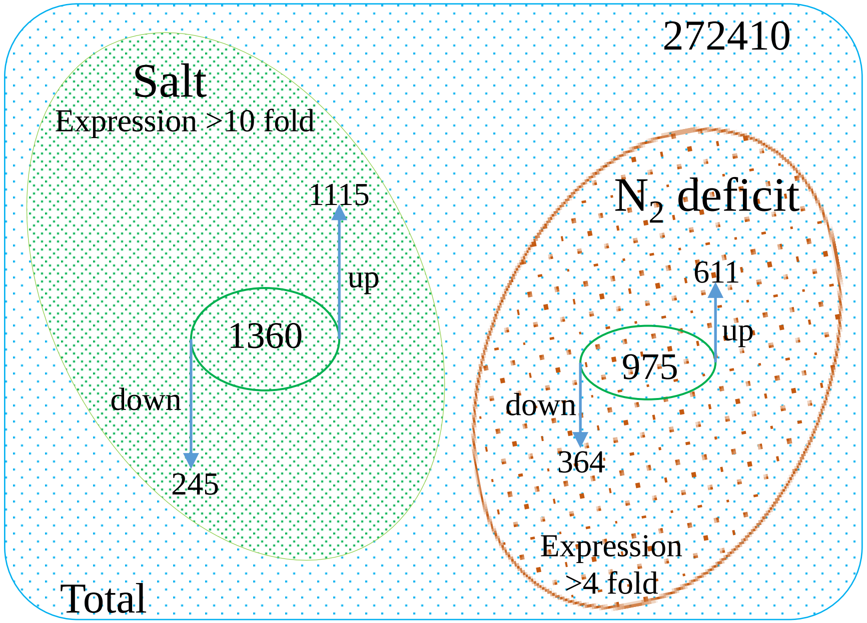

Figure S8: Venn diagram showing genes differentially expressed in *AhCytb6* transgenic plant compared to WT under nitrogen and salt stress condition as studied by microarray

Table S1: Primers used in the transcript profiling of differentially expressed genes/ clones obtained by Suppression subtractive hybridization (SSH)

Gene category	Primer ID	Primer sequences (5'→→→3')	Number of representing clones and individual clone id
Photosystem II_1	*Ah*PS F1	ACCAGCACTGAAAACCGT	**9** (357, 14,25,27,223,230,311,351,398,452)
	*Ah*PS R1	CCGCCGAAGTAGGAATA	
Hypothetical / uncharacterized_1	*Ah*HYP F3	ACTGTTAATAATCCCGCG	**12** (23,292,293,305,312,290,335,336,372,381,390,408)
	*Ah*HYP R3	CGTAGGTTTAGCAGTTTTAGA	
Photosystem II_2	*Ah*PS F4	TCAATAAAGTAGGGATCATCA	**4** (228,331,37,387)
	*Ah*PS R4	AAGATTTTACCATGACTGC	
Hypothetical / uncharacterized_3	*Ah*HYP F5	CTATTGCTTGTAACGTAGGTT	**3** (60, 271,274)
	*Ah*HYP R5	GTACCGAAACCATTAAAAGA	
Hypothetical / uncharacterized_4	*Ah*HYP F6	CGAGTCTTCATAGGGCAA	**5** (192,382,399,28,276,258)
	*Ah*HYP R6	GCTAGCTCTGGGTTCGA	
Hypothetical / uncharacterized_5	*Ah*HYPF7	CGAAACCATTAAAAGAACG	**2** (396,419)
	*Ah*HYP R7	GGTACTATTGCTTGTAACGTAG	
Hypothetical/ uncharacterized_6	*Ah*UNF1	CAAAGTCACCTGGAGCTG	**3** (220,345,365)
	*Ah*UNR1	AGCTTTCCCGTCTCTTCT	
Hypothetical/ uncharacterized_7	*Ah*UNF2	TACACGAATAGTTGATAGTTGAG	**3** (239,297,409)
	*Ah*UNR2	AAGATCGTTTGGCCATG	
Hypothetical/ uncharacterized_8	*Ah*UNF4	AGTCATAGTTACTCCCGCC	**3** (31,173,434)
	*Ah*UNR4	AGAACTGGTACGGACAAGG	
	*Ah*UNF9	CAGTGCTCCGAATGTCAA	**1**

Hypothetical/ uncharacterized_9	*Ah*UNR9	GTGGTTTCGCTGGATAGTA	(296)
Hypothetical/ uncharacterized_10	*Ah*HYP*F4*<br>*Ah*HYP*R4*	AGACCTCAGCCTGCTAACT<br>ACATCAGTGTAGCGCGC	**2**<br>(342,354)
Hypothetical/ uncharacterized_11	*Ah*HYP 8<br>*Ah*HYP 8	GGATACCTAGGCACCCAG<br>TGTTTCAGTTGGCCAGGT	**18**<br>(6,11,42,53,56,67,214,227,232,242,251,252,321,379,405,406,411,416)
Hypothetical/ uncharacterized_12	*Ah*HYP 9<br>*Ah*HYP 9	CTGGGGTTGAAGAAGGTC<br>CGCAGCAGTTCTTCCATA	**7**<br>(243,256,265,288,393,421,427)
Hypothetical/ uncharacterized_13	*Ah*HYP 10<br>*Ah*HYP 10	GTGAGACAGTTCGGTTCCT<br>CGCAGCAGTTCTTCCATA	**20**<br>(43,44,45,54,221,233,235,243,256,265,269,283,288,328,353,393,421,424,427,447)
Hypothetical/ uncharacterized_14	*Ah*HYP 11<br>*Ah*HYP 11	GGCATAACAACCGGTACA<br>GTTGTTCGCCGATTCAA	**4**<br>(278,281,306,348)
Ribulose bisphosphate carboxylase	*Ah*RB F1<br>*Ah*RB R1	GAAATCCTTGGGTGGCT<br>CGTTCAGGTCTGGCAAG	**7**<br>(57,177,178,189,318,423,428)

Table S2: Primers and PCR condition used in the study

Description of experiments	Primers code	Primer Sequences (5’→→→3’)	PCR condition
3’–RACE (rapid amplification of cDNA ends) to get full length cDNA towards 3’	SM 3’GSP-F1	TTGGCTAAAGGAATGGGT	As per instruction manual of RACE System for Rapid Amplification of cDNA Ends, version 2.0 (Invitrogen, Thermo Fisher, USA)
	SM 3’GSP-F2	CATCAATGATTGGGGAAC	
	SM 3’GSP-F3	CCATTTGCAACTCCTTTG	
5’–RACE (rapid amplification of cDNA ends) to get full length cDNA towards 5’	SM 5’GSP3-R1	GTTCCCCAATCATTGATG	
	SM 5’GSP3-R2	ACCCATTCCTTTAGCCAA	
	SM 5’GSP3-R3	GCAGGGAAATATCCAAATA	
Full length amplification of *AhCytb6* gene for cloning, molecular confirmation and quantitative reverse transcriptase PCR of transgenic lines	*Ah*Cytb6-F	ATGATTGGTTCGAAGAAC	[94°C 5 min] x 1<br>[94°C 5 min, 55°C 5 sec, 72°C 5 sec] x 30<br>[72°C 10 min] x 1
	*Ah*Cytb6-R	CTATAAAGGACCGGAGAT	
Amplification of *AhCytb6* gene for cloning in the intermediate vector pRT100	*Ah*Cyt-PCM-F	GGATCCATGATTGGTTCGAAGAAC	
	*Ah*Cyt-PCM-R	GAGCTCCTATAAAGGACCGGAGAT	
Reference/ housekeeping genes	uidA-F	GATCGCGAAAACTGTGGAAT	
	uidA-R	TGAGCGTCGCAGCAGAACATTAC	
	*Nt*Actin-F	CGTTTGGATCTTGCTGGTCGT	
	*Nt*Actin-R	CAGCAATGCCAGGGAACATAG	
